# Mechanical and Thermal Behavior of Fibrous Carbon Materials

**DOI:** 10.3390/ma14071796

**Published:** 2021-04-05

**Authors:** Blagoj Karakashov, M’Barek Taghite, Richard Kouitat, Vanessa Fierro, Alain Celzard

**Affiliations:** 1 Institut Jean Lamour (IJL), Université de Lorraine, CNRS, F-88000 Epinal, France; bkarakashov@gmail.com (B.K.); vanessa.fierro@univ-lorraine.fr (V.F.); 2 Institut Jean Lamour (IJL), Université de Lorraine, CNRS, F-54000 Nancy, France; mbarek.taghite@univ-lorraine.fr (M.T.); richard.kouitat@univ-lorraine.fr (R.K.)

**Keywords:** nonwoven, carbon felt, carbon fiber, compression, hyperelasticity, thermal conductivity

## Abstract

The ability of various commercial fibrous carbon materials to withstand stress and conduct heat has been evaluated through experimental and analytical studies. The combined effects of different micro/macro-structural characteristics were discussed and compared. Large differences in mechanical behavior were observed between the different groups or subgroups of fibrous materials, due to the different types of fibers and the mechanical and/or chemical bonds between them. The application of the Mooney–Rivlin model made it possible to determine the elastic modulus of soft felts, with a few exceptions, which were studied in-depth. The possible use of two different mechanical test methods allowed a comparison of the results in terms of elastic modulus obtained under different deformation regimes. The effective thermal conductivity of the same fibrous materials was also studied and found to be much lower than that of a single carbon fiber due to the high porosity, and varied with the bulk density and the fiber organization involving more or less thermal contact resistances. The thermal conductivity of most materials is highly anisotropic, with higher values in the direction of preferential fiber orientation. Finally, the combination of compression and transient thermal conductivity measurement techniques allowed the heat conduction properties of the commercial fibrous carbons to be investigated experimentally when compressed. It was observed that thermal conductivity is strongly affected under compression, especially perpendicular to the main fiber orientation.

## 1. Introduction

The competitiveness in many industrial applications of carbon felts (CFs), i.e., nonwovens based on carbon fibers, is due to the combination of unique structural and physical properties, giving them in particular extreme lightness together with good mechanical strength and excellent chemical and heat resistance. The main aspect to be considered when selecting a fibrous carbon structure for a given application is the level required for these materials properties. For example, the mechanical response of different nonwovens to transverse compressive forces plays an important role in almost all applications involving liquid infiltration processes for the preparation of composites [1], whereas the maximum chemical inertness is required for electrochemical uses [2,3]. Thus, in composite applications, the structure of the fibrous mat, which is used as a reinforcement, should be highly tortuous and suitable to accommodate the matrix and to withstand the infiltration process without being modified. At the same time, the fibrous host should improve/stabilize the thermal characteristics of the final composite and provide adequate long-lasting performance under isothermal or variable temperature conditions. As has already been studied [4], the mechanical properties of composites reinforced by carbon felts are also highly dependent on the distribution of heat in the bulk, thereby leading to or preventing internal stresses and micro-cracks.

Theoretically, changes in any of the raw materials and manufacturing steps should result in subtle differences in the structure and performance of the final fibrous carbon materials. Previous studies have examined the influence of differences between types [5,6] and properties of fibers [7,8,9,10] and manufacturing technology [11,12,13] on the compression behavior of nonwovens. It has been shown that the mechanical properties of nonwovens are strongly dependent on their structural characteristics. These include fiber morphology and type: fiber precursor, cross-section and diameter, crimp shapes of different fineness, modulus of elasticity and fiber denier. The mechanical properties also depend on the type of felt: fiber volume fraction, fiber orientation distribution, bonding technique. Nonwoven felts also exhibit various macroscopic mechanical responses depending on their manufacturing techniques, which influence the anisotropic distribution of the constituent fibers with preferential alignments/orientations and inter-fiber contacts/bonding/entanglements. Depending on the type of nonwoven, different mechanical behaviors can be observed [14]. If the structure is rigid (as in chemically bonded nonwovens), the stress-strain curve shows, after a short elastic linear zone, a permanent deformation typical of brittle materials. If, on the other hand, the structure is flexible [15] (as in needle-punched nonwovens, produced by depositing the fibers randomly on a flat support, forming a mat that is mechanically bonded by orienting a number of fibers perpendicularly by means of barbed needles), a very non-linear elastic deformation curve is observed [16]. These considerable differences in the shape of the stress-strain curves, typical examples of which are shown in Figure S5 of the Supplementary Material, are related to whether or not a gradual reorganization of the fibers, which may or may not be mobile depending on the type of consolidation of the mat, is possible during compression.

As previous studies on thermal conductivity have shown [17], the anisotropic bulk thermal properties of carbon fiber nonwovens are due to the different, themselves anisotropic, contributions of crystallites, fibers and the architecture of the felts on their own (see Figure S12 of the Supplementary Material). Indeed, even if the carbon from which the fibers are made is not necessarily graphitized, its nanostructure remains close to that of graphite. It is therefore very anisotropic, and so is its thermal conductivity. Since the carbon crystallites are preferentially oriented in the direction of the axis of the fibers, the thermal conductivity of the fibers is in turn very anisotropic. If the fibers are now preferentially oriented in the fibrous mat, the latter as a whole will have anisotropic thermal properties [15]. The thermal conductivity of carbon fibers is also directly related to structural defects, and thus to crystallinity, porosity, heteroelements and disorder in the orientation of crystallites [18,19,20]. As the temperature of the carbon fiber production process increases, the concentration of structural defects is reduced and crystal order is improved [21], which results in increased thermal conductivity. Recent studies on commercially available polyacrylonitrile (PAN)-based carbon fibers have shown that, at room temperature, the thermal conductivity of the fibers along their axis ranges from about 5 up to 156 W·m^−1^·K^−1^ [22,23]. The thermal conductivity values of carbon fibers based on regenerated cellulose (Rayon) along the axis have been found to be in the range of 5 to 15 W·m^−1^·K^−1^ [24]. Thermal conductivity can be used as a tool to characterize fibrous carbons prepared from various precursors under different manufacturing conditions. Morphological parameters which can have a great influence on the overall thermal conductivity are the aspect ratio of the fibers (length divided by diameter), the average fiber diameter, the number and nature of contacts between the fibers, and hence the felt consolidation technique, the porosity of the mat and the orientation of the fibers.

Since suppliers of fibrous carbons give numerical values for only a few of the physical properties of the materials they sell, such as weight per unit area, average thermal conductivity, felt thickness and carbon content (see Table S2 of the Supplementary Material), choosing a commercial carbon nonwoven for a particular application can be very difficult. The need to understand better the relationship between the manufacturing process and the final properties and to address the lack of data on their properties has led us to characterize and explore the properties of this type of materials. In particular, compression properties should be known prior to the preparation of composites [25]. Good compressive strength and recoverability are desired to maintain the initial structure of the fibrous preform. Therefore, the initial aim of this work was the experimental study of the mechanical properties of needle-punched CFs. A hyperelastic model approach was applied to predict some of the mechanical properties not directly measurable due to the large deformation of soft CFs. In addition to the standard mechanical testing technique, another non-destructive method was used and the data recovered from the two techniques were compared. The thermal conductivity properties of carbon nonwovens are also very important in many industrial applications. Here, we used the Hot Disk transient method, which allowed us to measure thermal conductivity values parallel and perpendicular to the directions of preferential fiber orientation of the commercial materials studied, whose properties are orthotropic. Finally, a simple device developed in the laboratory was used to examine thermal conductivity variations during compression. Note that, in view of the special (highly porous and disordered) nature of the samples, the terms “overall” or “effective elastic modulus” should be used instead of simply “elastic modulus”. The same applies to thermal conductivities, which are effective conductivities. However, in order not to make the text unnecessarily cumbersome, the terms “global” or “effective” will not be used systematically but should be implied in this article.

The results of these applied experimental and analytical studies are presented and discussed in this paper. To our knowledge, no other study has dealt so thoroughly with the properties of commercial fibrous carbons. This paper is the third of a series on the broad evaluation of the properties and use of such materials in new or improved applications [21,26]. The results obtained underline the importance of the mechanical behavior in compression and heat transfer that could influence the successful development, scale-up and industrialization of derived composites.

## 2. Materials and Methods

### 2.1. Commercial Fibrous Carbons Available

Samples of 18 commercial fibrous carbons (see Table S1 of Supplementary Material) were used here for the evaluation of their mechanical and thermal properties. In our previous studies [21,26], where the same materials have been studied for other properties and applications, we have already given the general characteristics of these materials, especially how they are manufactured, and how the precursor of the fibers and/or the production history can affect their structural and morphological properties at the micro/macro scale. It is particularly important to remember here that we are dealing with flexible CFs on the one hand, rigidized CFs on the other, and also rigid fibrous panels, and that their constituent fibers are either PAN-based (with two qualities: thin and thick fibers) or Rayon-based. Finally, all these materials were either carbonized or graphitized, depending on the heat treatment temperature seen by the fibers during their manufacturing process. Based on this general data, all materials were classified into groups and sub-groups, as justified in the first paper of this series [26]. This information, together with the names of suppliers, the trade names of the materials and the reference codes used in this work are given in Table S1 of the Supplementary Material.

Prior to any measurement on the materials, a preliminary desizing step was performed to guarantee the purity in terms of carbon content of the commercial materials, thus avoiding the risk of an additional and undesirable influence of the sizing layer possibly present at the surface of the fibers. For this reason, slow pyrolysis has always been applied to the samples [21], before any further examination of their physical properties.

### 2.2. Mechanical Characterization

#### 2.2.1. Classical (Destructive) Method: “Dynamic” Compression

A classical method for studying the compressive mechanical properties of materials is to apply an increasing load to them at a moderate and constant strain rate, and by measuring the resulting deformation. Using this method, stress-strain curves are obtained, from which different quantities can be derived. Despite its destructive nature, this technique is still the most frequently used for both soft and rigid fibrous materials [27]. It is normally described as quasi-static in the literature, but here we will call it “dynamic”, even if no acceleration effect is considered. This choice of terminology, which is strictly speaking incorrect, has nevertheless been made because the second technique for studying mechanical properties, discussed below and which is non-destructive, has been called quasi-static mechanical analysis (QMA) by its developers. It is therefore to avoid any confusion that the first technique, which is destructive because it involves considerable deformation of the samples, has been described as “dynamic”.

In the present case, the specimens were studied by axial compression using an Instron 5944 universal testing machine equipped with a 2 kN head (see Figure S6 of the Supplementary Material). The compression speed used was 0.5 mm min^−1^, following the ASTM D5729-97(2004)e1 standard [28] for the thickness measurement of nonwovens. During the compression test, stresses and strains were continuously recorded. Based on the corresponding curves, an attempt was made to estimate the modulus of elasticity and the yield stress. Indeed, when clearly observed, the slope of the initial, linear, elastic part of the curves defines the modulus of elasticity, *E* (MPa), within the limits of the application of Hooke’s law [29]:

(1) $$E\;=\;\frac{\sigma }{\epsilon },$$

where $\sigma \;=F/S_{0}$, expressed in MPa, is the uniaxial non-zero stress, *F* (N) being the compression force applied to a defined cross-section *S*_0_ (m^2^), and where *ϵ* (dimensionless) is the strain, i.e., the relative deformation (change in sample thickness, ∆*L*, divided by the original thickness of the mat, *L* (mm)). The yield stress (MPa), also called the elastic limit, has been defined as the maximum stress in the elastic region: it thus corresponds to the end of the elastic behavior and the beginning of the plastic behavior.

**Figure 1:**
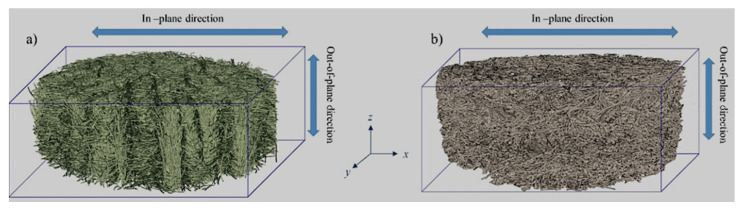
Realistic 3D representation of different fibrous carbons, obtained from tomography analysis: (**a**) soft felt and (**b**) rigid board samples, with indication of *xy*-direction (i.e., in-plane “IP” direction) and *z*-direction (i.e., out-of-plane “OP” direction).

Three cylindrical samples of diameter 44.45 mm (1 ¾ inch) and a thickness measured according to the ASTM D5729-97(2004)e1 standard [28] by use of the same Instron 5944 [26], were tested in the out-of-plane (OP) direction (see Figure 1) for each kind of material.

#### 2.2.2. Non-Destructive Method: Quasi-Static Mechanic Analysis

A non-destructive technique, based on the damping of mechanical vibrations, was also examined in the case of soft CFs. These materials indeed present mechanical properties that can vary from purely to partly elastic over the entire range of deformation, depending on their manufacturing process.

The QMA is a rather recent and uncommon method for determining the elastic modulus of porous materials. The process of measuring the properties of poroelastic materials by this technique is explained in detail elsewhere [30]. Briefly, it is based on the polynomial relations between compression stiffness, elastic modulus, Poisson ratio and shape factor, and the estimation of the latter parameters can be obtained from high-order axisymmetric finite element simulations using a disc-shaped sample under static compression (see Figure S9 of the Supplementary Material). The shape factor is defined as half the radius to thickness ratio of the sample. The relations established operate in the low-frequency range, below any resonance, and take into account the fact that the disc-shaped sample may swell laterally when compressed between two rigid platens.

The procedure for performing QMA tests is detailed in Section 3.2 of the Supplementary Material. The quasi-static compression test was performed on at least three samples of each material. CF specimens of various thicknesses (imposed by the manufacturer), ranging from 9 to 20 mm, and a single diameter of 44.45 mm, were therefore analyzed.

### 2.3. Thermal Conductivity Characterisation

The Hot Disk technique is a transient plane source method for the rapid measurement of the thermal conductivity of solid, granular or paste-like materials and even liquids. The basic theory behind its principle has been discussed elsewhere [31]. Briefly, this method is based on the transient heating of a flat double spiral inserted between two pieces of the material under investigation (see Figure S13 of the Supplementary Material). From the temperature rise of the heat source, and provided that the total duration of the measurement is chosen within a correct time window defined by the theory and the experimental conditions, thermal conductivity and thermal diffusivity can be obtained from a single experiment.

Thermal properties are strongly influenced by the anisotropy of the materials (both at fiber and nonwoven scale), which makes it difficult to measure them exhaustively using conventional instruments (hot-wire and hot-strip, flash method, guarded hot plate, etc.). Indeed, most of them give either the conductivity along only one direction, or an apparent overall conductivity. On the other hand, on the condition that the volume heat capacity of the material is known, the transient plane source method can be applied to the case of anisotropic materials. In the case of the present carbon nonwovens, the thermal properties along two of the orthogonal and main axes (*xy*–in-plane) are considered to be identical (IP thermal conductivity, *κ_xy_*), and therefore different from those along the third axis (*z*—out-of-plane) (OP thermal conductivity, *κ_z_*) [32].

The measurement protocol and procedure are explained in Section 4 of the Supplementary Material. The thermal conductivity was measured after the desizing step, and at least 3 replicates of different samples from the same material were carried out. The measurements were performed with the isotropic and anisotropic modes of the measuring device and with or without applied compression, in order to compare the values and discuss the influence of the material structure.

## 3. Results and Discussion

### 3.1. Mechanical Properties of Commercial Fibrous Carbons

The objective of this part was to study the mechanical properties of porous nonwoven carbon materials as a function of their structural characteristics. Thus, the capacity of commercial fibrous carbons to withstand the stresses applied in the OP direction was evaluated by two different characterization methods, in order to compare the recovered data.

#### 3.1.1. Destructive (“Dynamic”) Compression Method

**Figure 2:**
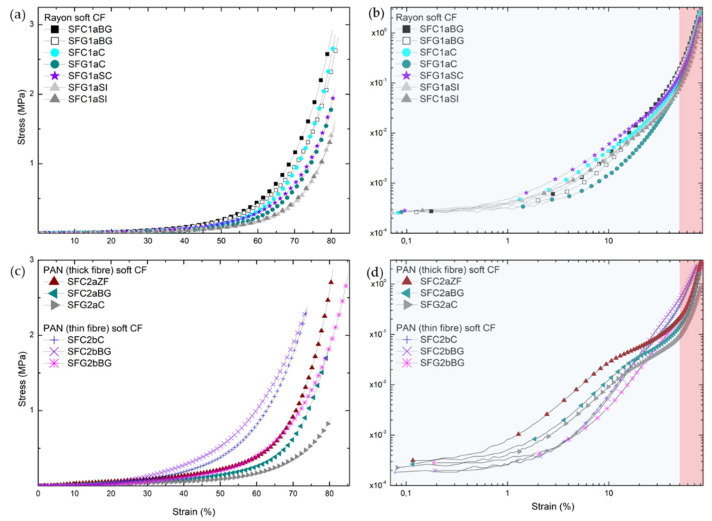
Stress-strain characteristics of soft carbon felts (CFs), subjected to uniaxial compression along their out-of plane (OP) (z) direction, derived from: (**a**) Rayon-derived and (**c**) polyacrylonitrile (PAN)-derived (thin and thick fibers); Logarithmic representations of (**a**) and (**c**) are given in (**b**) and (**d**), respectively, with indication of the compression zones influenced by different deformation regimes (“non-linear elastic”—transparent blue and “solid”—transparent red zone).

The stress-strain curves of all soft carbon felts (CFs) are shown in Figure 2. The deformation of most of these materials (see Figure 2a,c) is highly non-linear over the entire range of applied stress. Their mechanical properties should be governed by reversible mechanisms (stretching and rotation of the fibers) as well as irreversible mechanisms (sliding and/or interlocking of the fibers, breakage of the fibers and the bonds between them and/or disentanglements). Indeed, when performing these uniaxial compression experiments, we found that the soft CFs did not completely return to their original dimensions when compressed to more than 70% of the initial thickness, suggesting that the non-linear behavior is influenced by plastic deformation. Thus, this mechanical response corresponds to that of randomly cross-linked elastic fiber networks with limited irreversible extensibility and compressibility. As has been suggested elsewhere [33], this class of elastic fibrous materials encompasses nonwovens as well as elastomers and biological materials with macromolecular components such as elastin and collagen.

The logarithmic presentation of the stress-strain results suggests that at least two compression regimes govern the deformation of soft CFs. These consecutive deformation regimes are important characteristics addressed by several previous studies [1,34,35]. First, non-linear elastic deformation (indicated by the transparent blue zone in Figure 2b,d) of soft CFs is due to reorientation, sliding, stretching and/or nesting of the constituent fibers in the low to moderate deformation range, i.e., until the porosity decreases down to typically 50%, depending on the material. As observed in previous SEM analyses [26], the fibrous layers oriented in the IP direction, positioned one on top of the other, show very little contact between the fibers, in accordance with the high porosity of these materials. Because of such deformations, the fiber layers in the IP direction are compacted, which narrows the pores between them. Thus, a minimal stress response is observed under the influence of an external uniaxial load. An increase in stress response in this regime is only observed for soft (thin fibers) PAN-derived CFs, which are the ones in the study with the highest needle-punching density [26]. The latter implies that the fibers in the OP direction give to these materials a higher stiffness compared to other groups of soft CFs. At higher strain, the interfibrous void space is almost entirely occupied by carbon fibers, giving the felt a much more compact structure that prevents further elastic deformation of the fibers. Therefore, the non-linear elastic deformations almost completely stop and the constituent fibers are finally deformed by irreversible plastic mechanisms (transparent red zone in Figure 2b,d). This compression regime implies a continuous and irreversible change in the structure of the felt. Moreover, higher and higher stresses, which increase very rapidly, are required to continue to deform the material as the friction between the fibers intensifies and the fibers become compacted. The two proposed deformation regimes lead to an increase in the stiffness of CFs subjected to increasing loads, due to the complex non-linear elastic and then plastic evolution of flexible CFs.

Fiber orientation and felt bulk density influence the uniaxial compressive mechanical properties of all soft CFs studied with the conventional method, and their effects are detailed below:

- Effect of fiber orientation on compression properties

Different fiber orientation distributions have been observed in CFs manufactured by different needle-punching processes in terms of needle-punching density, penetration depth and needle type, as also reported in previous studies [27,36,37]. As Karakashov et al. indicated [26], fiber reorientation in the soft CFs studied is induced during this needle-punching operation: fibers initially arranged randomly in the IP direction are pushed by the barbed needles in the OP direction.

**Figure 3:**
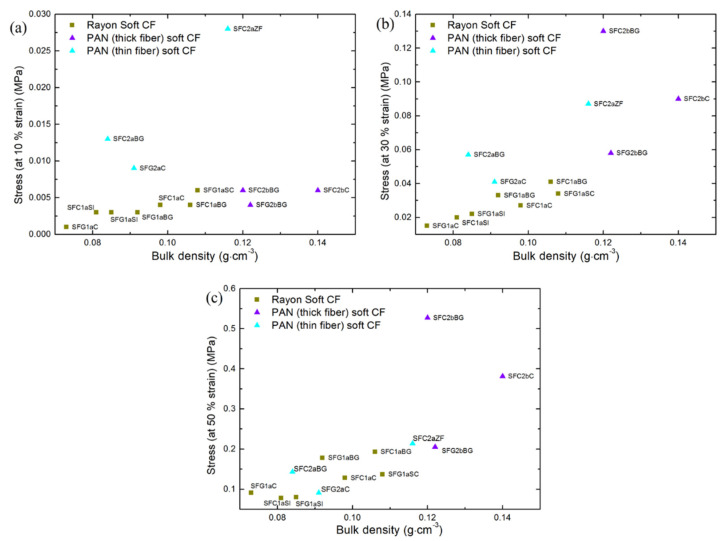
Effect of bulk density on the stress applied to CFs for deforming them at: (**a**) 10% strain; (**b**) 30% strain and (**c**) 50% strain.

Herein, the averaged fiber orientation was studied quantitatively by micro-computed tomography (µ-CT) analysis (see Section 2 of the Supplementary Material). This structural analysis was considered useful to discuss the effect of fiber orientation on the compression properties of CFs. Based on the µ-CT analysis (see Figures S3 and S4 of the Supplementary Material), a higher needle-punching density and penetration depth should have resulted in an increased fiber orientation in the OP direction, observed only for PAN-derived thin-fiber CFs. Therefore, the increased fiber density in the OP direction resulted in CFs with a higher stress response, such as SFC2aZF, SFC2aBG and SFC2aC, even at a strain less than 20%. This characteristic has not been observed in materials of the other groups of soft CFs of similar bulk densities (see Figure 3), for which the stress responses up to 30–50% strain are the result of the reorganization and bending of the fibers, and not of the compression at fiber-fiber contacts. In other words, at low compression strain, the fibers in the OP direction first bend (giving the elastic limits of the material), then tend to reorient in the IP direction and finally, during solid-state compression, are plastically deformed. Therefore, they change from elastic to plastic deformation once the stress is higher than 30–50%. During solid-state compression, almost all fibers should be unidirectional in soft CFs, since most OP fibers are already reoriented in the IP direction.

- Effect of felt bulk density on compression properties

As shown in Figure 3, the compression stresses of all the soft CFs, whose values are shown in Table S4 of the Supplementary Material, increases with their bulk density. When considering Rayon-derived soft CFs, this trend is the most obvious and consistent, considering the increase in strain from 10 to 50 %. µ-CT analysis clearly shows that most CFs derived from Rayon have a similar fiber orientation (see again Figure S3). If similar distribution of fiber orientation is considered, it is very likely that differences in fiber morphology (fiber cross-section and/or crimp) and density of fibers in the IP direction will influence the changes observed from the bulk density. Therefore, increasing bulk density contributes to improving compression stiffness. It should be expected that the contributions of fiber and felt properties and density would be much greater and clearer if CFs were made from the same material and with the same manufacturing process, and by changing only one fiber parameter at a time.

**Figure 4:**
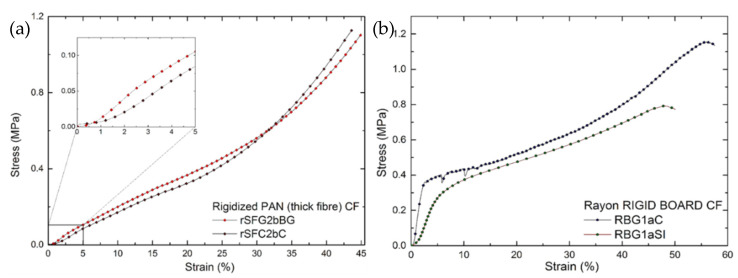
Stress-strain characteristics of: (**a**) rigidized PAN-derived soft CFs and (**b**) Rayon-derived rigid boards, subjected to uniaxial compression along their OP (*z*) direction. The inset in (**a**) is a zoom on the initial linear deformation.

For rigidized, PAN-derived, soft CFs, a higher compression stress is observed at much lower strain, compared to the previous groups of soft CFs. Differences in manufacturing process, such as the rigid bonds introduced on the upper and lower surfaces of initially soft felts, and between two or more sheets needle-punched on top of each other along the OP direction [26], prevented the elastic deformation observed in soft CFs. Thus, bonding the surface fibers with a rigid matrix should contribute significantly to the mechanical properties by reducing the probability of free fiber movement. Consequently, when uniaxially compressed, these materials clearly exhibit a linear elastic part before being deformed and densified in a non-linear manner (see Figure 4a). The elastic modulus was then simply calculated using Equation (1) (see Table 1). Beyond the initial linear part, the fixed connections between the fibers and the binder must be continuously destroyed, freeing the fibers to rearrange until complete fiber-fiber solid-state compression occurs. Thus, the materials are irreversibly deformed after the elastic deformation limit. The value of the yield stress (elastic limit) was considered as the stress up to which the materials can be elastically deformed and can reversibly return to their original dimensions. The strength of these materials increased with the bulk density, mainly due to the increase in binder concentration [38].

Figure 4b represents the stress-strain curves in the OP direction of rigid boards derived from Rayon and manufactured from chemically bonded chopped fibers. Table 1 presents the elastic modulus calculated from Equation (1), and the yield stress values. These materials deformed linearly at a much lower strain, less than 2%, and then the stress increased non-linearly with strain, as did the compression deformation of previously studied chemically bonded chopped fiber structures [16]. For the two rigid board samples studied, a constant non-linear deformation was observed after the yield stress. Beyond the latter, the material started to deform plastically, i.e., it exhibited permanent deformation due to the collapse and disintegration of the bonded fiber layers in the OP direction. These observations separate the rigid boards from the rest of the soft CFs and rigidized soft CFs, for which the non-linear deformation is mainly due to fiber rearrangements.

In contrast to soft CFs, where the distribution of fiber orientations has a major effect, the mechanical properties of rigidized soft CFs and rigid boards depend mainly on the bond strength at the interface between the fibers and the carbon binder. The phenomenon of binder distribution and accumulation at the fiber intersections contributes, as expected, to the overall mechanical properties of the materials. As previously reported, this is due to the increase in effective load transfer between the fibers when exposed to external compression or other mechanical forces [38]. Therefore, the elastic modulus and strength values of the RBG1aC samples were higher than those of RBG1aSI, mainly due to the increased binder concentration (deduced from the increased density of the felt) and the improved interface bond between the binder and the carbon fibers. Taking into account the differences in the manufacturing technique, we can also observe that rigidized soft CF samples, with a lower binder concentration and only at the periphery [26], have a much lower modulus and strength than rigid board samples, with evenly distributed regions of fiber-matrix connections.

In the case of rigidized soft CFs and rigid boards, we have considered the materials evaluated as initially linear elastic or Hookean, i.e., where the force required to compress them to a certain thickness is proportional to that thickness [39]. In contrast, the compression of soft CFs involves large, essentially non-linear deformations, and the corresponding stresses depend on the basic properties of the fiber/felt. For soft CFs, the mechanism of free, continuous and complex fiber deformation mainly results in continuous non-linear deformation, corresponding to low compressive stresses and compression at fiber-fiber contacts only starting at high deformation. Consequently, the stress-strain experimental results suggest the hyperelastic nature of soft CFs. Moreover, the mechanical properties of these non-linear elastic materials cannot be directly represented by concepts of linear elasticity, but by constants defined from experimental functions of the non-linear deformation [40].

This compression behavior of fibrous nonwovens was first studied by Wyk [41], who considered fiber bending and the number of contacts between fibers as the main parameters for modeling their mechanical behavior. Subsequently, Wyk’s empirical solutions were modified by many authors to take into account the felt structure and the elastic/irreversible fiber-to-fiber effects. The objective of our study was not to develop new analytical solutions for the non-linear behavior of soft CFs, but to analyze their compression behavior using an existing analytical model and to compare the experimental results obtained. For a simple and practical application, a modified hyperelastic model with few parameters and constants coefficients was examined. In this way, the obtained model solution was directly related to the linear elastic constitutive parameters of interest. Thus, the values derived from the measurements were fitted with an empirical hyperelastic model for determining important information that is not directly measurable, such as the elastic modulus, due to the absence of a linear region in the stress-strain curves of softs CFs.

As described earlier [33], the soft CFs studied here can be defined as networks of randomly interconnected fibers, resembling the microstructure of elastomers based on randomly cross-linked polymer chains. Following the same concept has allowed us to apply a hyperelastic material model, commonly used to describe the behavior of rubber [42], to the study of soft CFs. Thus, the Mooney-Rivlin model [43,44] is widely accepted for investigating the elastic behavior of rubber-like materials up to 200% tensile strain [45,46].

The elastic modulus is estimated in practice simply by fitting the Moonley-Rivlin model to the experimental data [47]. When studying samples of nonwoven fibrous materials whose diameter is smaller than that of the compression platens of the device, the deformation is perpendicular to the plane and lateral expansion of the material, in the IP direction, is not expected due to their intrinsic nature. The Poisson ratio, being the ratio between transverse and axial deformation, is indeed assumed to be close to zero [48,49]. Herein, the value of this parameter is considered 0, not only because of the information found in the literature but also because of the QMA tests performed, presented in the next Section 3.1.2.

Hyperelastic materials can be described under the assumption of the existence of a deformation energy potential [46]. In the application of the Mooney-Rivlin model to soft fibrous materials with zero lateral deformation, one can write:

(2) $$\lambda_{z}\;=\;1\;+\;\frac{∆L}{L},$$

where

∆L/L is the dominant longitudinal OP deformation, i.e., along the *z*-axis. With a known density of strain energy, it is possible to determine the dependence of the nominal stress on the strain. Therefore, using the compressive stress $F/\;S_{0}$, where *F* is the force applied on the sample of cross-section *S*_0_, the modified Mooney-Rivlin equation reads:

(3) $$\frac{F}{{2\;S}_{0\;}{(\lambda }_{z}\;-\;\frac{1}{\lambda_{z}^{2}})}{\;=\;C}_{10}\;+\;\frac{C_{01}}{\lambda_{z}}$$

The left-hand side of Equation (3) represents the resultant stress and the right side is linear with respect to the variable $1/L\lambda_{z}$, which allows the Mooney-Rivlin constants *C*_10_ and *C*_01_ to be determined. Therefore, a numerical function was created for the simultaneous identification of the two Mooney-Rivlin constants, using Equation (4):

(4) $$\frac{F}{S_{0}}\;=\;2\left(1\;-\;\frac{1}{{1\;-\;\lambda }_{z}^{3}}\right){[(1\;-\;\lambda }_{z}{)C}_{10}{\;+\;C}_{01}]$$

Fitting the model to the experimental stress-strain curves of the soft CFs (see Figures S7 and S8 of the Supplementary Material) allows the two Mooney-Rivlin constants to be identified (see Table 2). As presented earlier [40,50], the Mooney-Rivlin constants obtained can be correlated with the Lamé constant, *µ*, which has the same meaning as the nonlinear shear modulus, by Equation (5):

(5) $${2\;(C}_{10\;}{+\;C}_{01})\;=\;\mu$$

Finally, a single value of the elastic modulus, E*, can be defined as twice the shear modulus by the relationship:

(6) E* = 2μ,

considering only the dominant OP deformation. The resultant values of the elastic modulus are presented in Table 2 for each of the soft CFs evaluated here.

**Figure 5:**
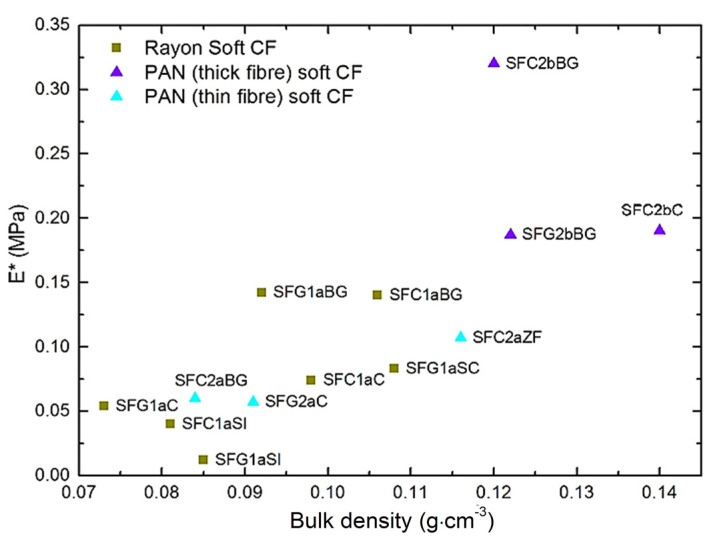
Elastic modulus values of soft CFs, calculated from Equation (6), as a function of bulk density. The symbols have the same meaning as in Figure 3.

When the values of elastic modulus obtained from Equation (6) are plotted against bulk density, see Figure 5, most soft CFs show similar trends to those seen in Figure 3. However, the differences observed in Figure 3 between soft CFs derived from PAN (thin fibers) and those derived from Rayon have disappeared, and both groups now show comparable modulus at a given bulk density. The calculated elastic modulus values, E*, for PAN-derived (thin fibers) CFs are lower than expected, and this is the result of the model’s assumption of purely non-linear material deformation, with rubber-like properties. Yet, the tested PAN (thin fibers) nonwovens exhibit somewhat different elastic fiber deformations. Therefore, as presented in Figure S8b of the Supplementary Material, and whereas the fit looks excellent, the model slightly underestimated the experimental stress up to the beginning of the solid compression regions (above 50% of porosity), which lowered the values of the Mooney-Rivlin constants. The opposite is observed by fitting the experimental stress-strain curves of the soft CFs derived from Rayon and PAN (thick fibers) (see again Figures S7 and S8a). These differences in model fit are due to differences in fiber layer formation and fiber orientation distribution. In particular, the soft CFs derived from PAN (thin fibers) are those having the highest proportion of fibers oriented in the direction of compression (OP direction).

**Figure 6:**
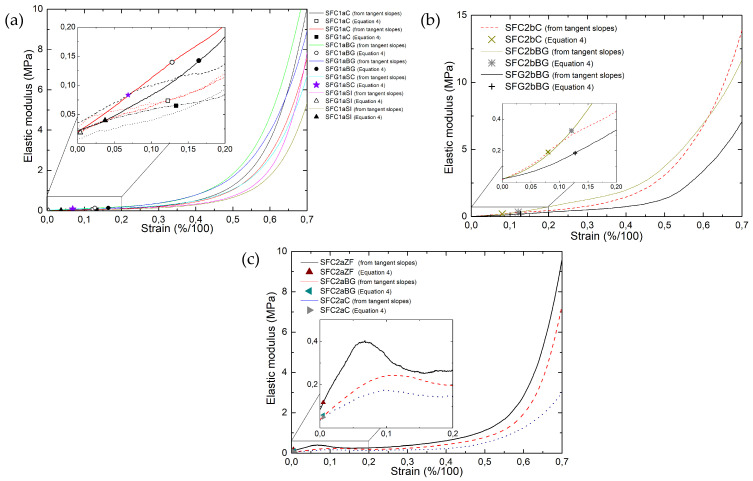
Elastic modulus (E*) calculated by Equation (6) (symbols) and slopes of the tangents to the experimental stress-strain curves (lines) for soft CFs derived from: (**a**) Rayon; (**b**) PAN (thick fibers) and (**c**) PAN (thin fibers).

We saw that the particular shape of the stress-strain curves (Figures S7 and S8) leads to an ill-defined elastic modulus since no linear zone exists but only local values of the slope at different stresses can be given, as was done in Figure 3. On the other hand, and this is its interest, Equations (4)–(6) give by fitting to these same curves a single value of elastic modulus for each CF. The corresponding values are given as discrete symbols in Figure 6, where they are compared with the slopes of the stress-strain curves, which vary continuously. For soft CFs derived from Rayon (thick fibers) and those derived from PAN (thick fibers), these moduli correspond to the slopes of the stress-strain curves at very low strain, but not as much as those found for thin-fiber CFs derived from PAN, corresponding to the very beginning of the deformation of these materials (see Figure 6c).

The excellent correlation between the modulus values determined by Equation (6) for carbonized and graphitized felts from the same precursor, such as SFC1aC↔SFG1aC, SFC1aBG↔SFG1aBG and SFC1aSI↔SFG1aSI (see Figure 5), is probably related to their structural similarity. In contrast to the other two groups of soft CFs, PAN-derived thin-fiber CFs show a significant (linear) increase in stress at low strain (between 5 and 10%, Figure 6c). This differs from the continuous exponential increase of modulus at each different strain observed for the other two CF groups. Therefore, the difference in fiber orientation and compressive properties makes the hyperelastic model unable to predict the correct value of the single elastic modulus of PAN-derived thin-fiber CFs. The different compression behavior of the PAN-derived (thin fibers) CFs is observed due to the high proportion of fibers oriented in the OP direction, deduced from the μ-CT analysis (see again Figure S4a). Due to these structural characteristics, the compression of these materials is initially dominated by elastic bending deformation of the fiber bundles in the OP direction. The elastic deformation of the OP fiber bundles thus leads to a higher compression stress than that of the CF samples derived from Rayon and PAN (thick fibers).

**Figure 7:**
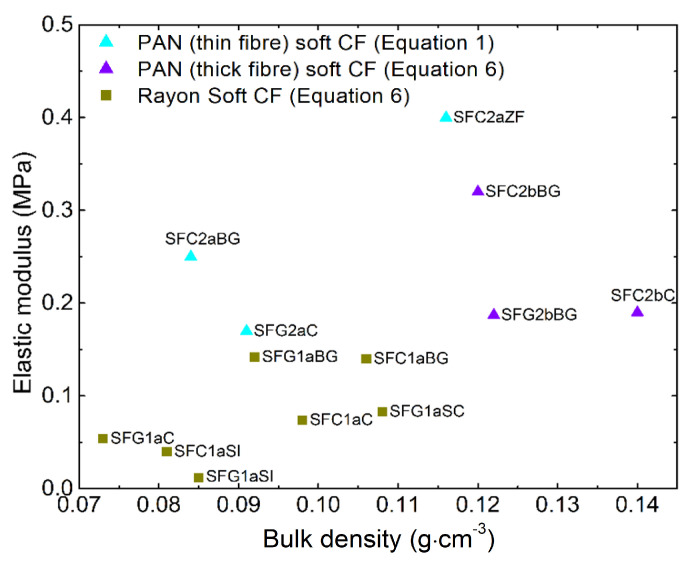
Values of elastic modulus calculated either by Equation (1) for soft CFs derived from PAN (thin fibers) or by Equation (6) for soft CFs derived from Rayon and from PAN (thick fibers), as a function of bulk density. The symbols have the same meaning as in Figure 3.

The slope values of the initial parts of the stress-strain curves are shown in Table S5 of the Supplementary Material for CFs derived from PAN (thin fibers), obtained by applying Equation (1) to the linear part observed at very low strain. These values are shown in Figure 7 as a function of bulk density, where the moduli of the CFs derived from rayon and PAN (thick fibers) obtained by Equation (6) are also shown. This result strongly suggests that the elastic modulus of soft CFs can be evaluated using Equation (1), implying linear behavior at low strain, or through parameters that are explicit functions of the deformation with Equation (6), which applies over the whole range of deformations. Therefore, it can be accepted that for materials with continuously non-linear deformation, a solution for the elastic modulus solution can be found with the use of the Mooney-Rivlin hyperelastic model, even if the fit slightly underestimates the elastic deformation of the materials. Whereas in the case of the PAN-derived (thin fibers) soft CFs, the presence of initial regions of linear elastic deformation leads to lower modulus estimates with the Mooney-Rivlin model, and thus the use of Equation (1) is more accurate. The latter experimental results and modulus calculations are supported by uniaxial compression studies of woven fabrics, where the experimental stress-strain curves also show an initial linear part before the non-linear deformation [51,52,53]. The comparison between soft CFs derived from PAN (thin fibers) and woven fabrics is acceptable since in both cases the observed initial linear part of the compression curve should be the result of bending deformations of the fibers with increased compression resistance under the applied load.

#### 3.1.2. Non-Destructive Method: Quasi-Static Mechanic Analysis

All soft CFs were investigated not only with the aforementioned destructive method, but also by a quasi-static, non-destructive method. Soft, porous materials are sometimes characterized by QMA to initially identify the Poisson ratio, allowing the elastic modulus and damping loss factor to be calculated [30]. However, in the present case of highly porous materials with weak and loose fiber connections, the absence of lateral deformation (i.e., in the IP direction) during the characterization process makes it possible to carry out measurements with an imposed zero value of the Poisson ratio. As observed earlier, this fixed value of the Poisson ratio can be correctly used to directly calculate the actual elastic modulus and loss factor (parameter that defines the ability of a material to convert part of the acoustic, vibration, energy into heat by sound attenuation) of soft fibrous materials [54,55].

**Figure 8:**
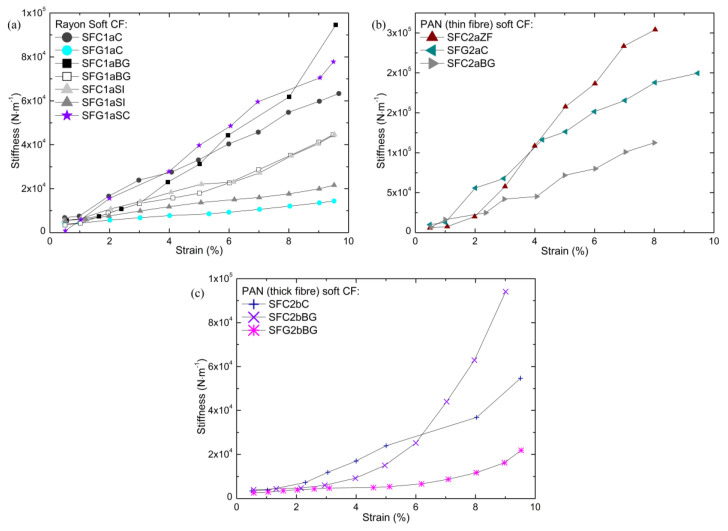
Stiffness vs. strain measurements for the subgroups of soft CFs derived from: (**a**) Rayon, (**b**) PAN (thin fibers) and (**c**) PAN (thick fibers).

As explained in Section 2.2.2, the choice of the appropriate measurement parameters such as sample geometry, compression strain and excitation frequency, is very important for the correct determination of the vibro-acoustic performance of soft CFs. The measurements of the stiffness as a function of strain, performed to help identify a proper pre-compression deformation, are shown in Figure 8. The results show that no plateau like the one shown in Figure S10 of the Supplementary Material (Stage 2) is identified. As in dynamic compression tests, these results show a strong dependence of the elastic properties on the strain used, with soft CFs becoming stiffer with increasing static preload. Given the constant increase in stiffness with strain, the range of deformations used in the characterization tests must therefore be always specified when analyzing fibrous materials. Here, soft CFs were preloaded with a strain of 1–3% before the elastic modulus and loss factor were measured. The range of deformation selected was that at which the stiffness of the materials was similar in both compression methods, comparing the morphology of the present CFs with that of other fibrous materials analyzed in a previous work [56]. The excitation frequency was also fixed and identical in each measurement, as it had previously been observed to affect the elastic properties of fibrous materials, in addition to the effect of the pre-compression load.

**Figure 9:**
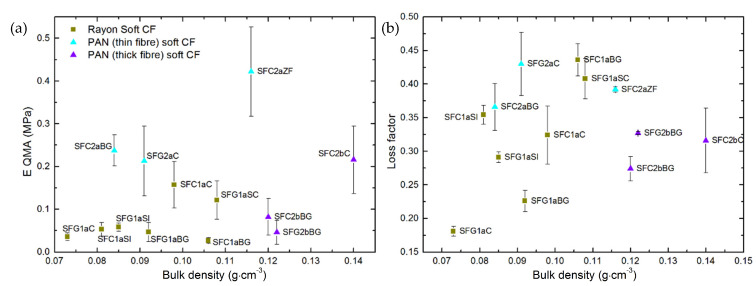
Elastic and damping parameters obtained by quasi-static mechanical analysis (QMA) as a function of bulk density: (**a**) Elastic modulus and (**b**) loss factor. The symbols have the same meaning as in Figure 5.

Table S6 of the Supplementary Material gathers all the results of the measurements of elastic modulus and loss factor of the soft CFs obtained by the QMA method. By plotting the values of elastic modulus (see Figure 9a) as a function of bulk density, the same effect of the structural characteristics of soft CFs on their elastic properties can be observed as seen with the dynamic compression method. Although a general trend is difficult to observe, the elastic modulus of each sub-group globally increases with density. Again, PAN-derived (thin fibers) soft CFs give the highest elastic modulus compared to samples derived from Rayon and PAN (thick fibers) over the wide range of densities considered. Changes in elastic modulus are again observed due to the different proportions of fibers reoriented from the IP to the OP direction during the needle-punching process, as shown by SEM and tomographic characterization. Concerning all PAN-derived soft CFs, the decrease in fiber diameter and/or the increase in fiber density in the IP direction resulted in a higher modulus of elasticity due to the improved contact between the fibers. Nevertheless, these observations would have been clearer if it had been possible to analyze CF series in which only one manufacturing parameter or one material property was changed at a time, which was not possible with the available (already very wide) series of commercial CFs.

The damping loss factor of the soft CFs was also obtained by QMA measurements (see Table S6 and Figure 9b). The loss factor was found to be in the range of 0.181 to 0.436, and generally tends to increase with the bulk density of the materials. Moreover, the loss factor is seen to be density-dependent when observed within individual families and subgroups of soft CFs, which is particularly evident for Rayon-derived materials, although the results are scattered. Damping in porous, fibrous, fluid-saturated media, i.e., the reduction in the amplitude of oscillation due to energy loss of the system to overcome some resistive force, results from two different mechanisms. One of these mechanisms is due to the viscous drag at the interfaces between the surface of the fibrous material and the fluid moving relative to the felt. The other is associated with the deformation of the solid fiber network. As previously observed [57], both mechanisms coexist for the materials studied under atmospheric pressure. Therefore, the inhomogeneous cross-section of Rayon-derived CFs may cause higher viscous drag. The latter should have led, for most Rayon-derived CFs, to loss factors higher than, or similar to, those of PAN-derived (thin fibers with circular cross-section) CFs of the same or higher bulk density. In the case of PAN-derived (thick fibers, also with circular cross-section) CFs, the loss factors are low even at high bulk density, due to the increased fiber diameter and voids in the IP interlayers compared to other soft CFs made of thinner carbon fibers. Using QMA, we obtained the loss factor at a fixed frequency of 20 Hz. Normally, this quantity is investigated in acoustic insulation applications of this type of materials and is studied in a higher frequency range of 100–5000 Hz [58]. Therefore, these results could be considered as preliminary information for future research to evaluate new applications for commercial CFs.

#### 3.1.3. Comparison of Destructive and Non-Destructive Methods

Information on the intrinsic mechanical parameters of CFs is crucial for a prediction of their performance that is more accurate, e.g., in composite preparation, vibro-acoustic applications and many others. This is why, using the broad variety of soft CFs, we have attempted to compare directly the results of the two methods used to determine the elastic modulus. The novelty is to provide important information on the accuracy and/or dispersion of the elastic properties of soft CFs, measured with either a dynamic and destructive method or a quasi-static and non-destructive method.

**Figure 10:**
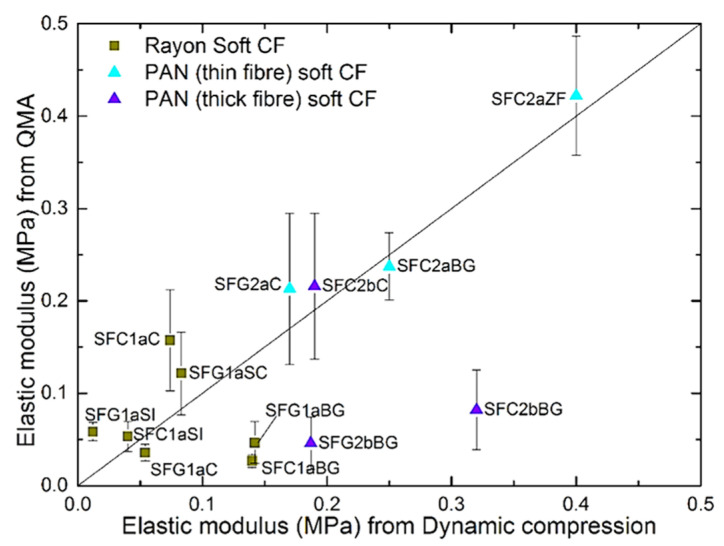
Comparison of modulus of elasticity values by QMA method and by dynamic compression method.

The elastic moduli calculated by QMA were plotted against those obtained by the dynamic compression method. This is shown in Figure 10, where a diagonal straight line has been added to guide the eye. In general, many values are the same or very close for both methods, except for the samples SFC1aBG, SFG1aBG, SFC2bBG and SFG2bBG. These materials come from a single supplier, and even if they are derived from different precursors, they should have been manufactured according to similar protocols. Thus, these materials have significantly different structural and mechanical characteristics from those of the other CFs examined. Moreover, using a compression strain between 5 and 6% gives elastic modulus values for the SFC1aBG, SFG1aBG, SFC2bBG and SFG2bBG samples by the QMA method closer to those obtained by the dynamic mechanical method (see Figure S11 of the Supplementary Material). This could be due to a different spatial rearrangement of the constituent fibers under compression, and fewer additional fiber-fiber contacts occurring up to the same strain, compared to other soft CFs. The choice of the strain at which the modulus is calculated must therefore be adjusted according to the demanded mechanical properties of the CFs in their final application. For this reason, the investigation of the elastic modulus of soft CFs by the QMA method must be supported by information on the strain at which the tests were performed, especially when comparing the elastic behavior of a given material using different techniques. 

Although destructive, the dynamic compression method is more suitable for determining a global elastic modulus, using Equation (6) for hyperelastic materials or Equation (1) for materials with an initial linear response under constant load increase. In contrast, QMA is only suitable for determining the elastic modulus of soft CFs at a defined pre-compression load, without giving the global elastic modulus of the materials.

When working with highly compressible materials, it is very important to know the value of the force at which the initial thickness of the materials was measured, before carrying out compression tests. In measurements made with the dynamic compression method, the initial contact was obtained at a predefined force value, as explained in Section 2.2.1. On the contrary, in mechanical analysis with the QMA method, the "initial contact" is estimated using the value of the nominal force, but also the rate of its change to avoid any false negative movement. It is therefore difficult to deduce an absolute value of the force at which the initial contact was recorded. The comparison of the results obtained with the QMA and any other technique must therefore be carried out with great care, as the initial thickness of the soft CFs may vary from one test to another (using the same method as in the case of QMA) or from one mechanical compression method to another.

The results obtained also indicate which of the two methods is the most appropriate for studying the elastic properties of soft CFs. If the materials are used for static purposes, where creep is expected under persistent mechanical load, the study of the materials should be carried out by the dynamic mechanical compression method. Otherwise, the materials should be examined by QMA in a vibro-acoustic application. Moreover, it has been previously observed that the QMA method is suitable for measuring the elastic modulus of viscoelastic materials only in the absence of brittle mechanical deformation [30].

### 3.2. Thermal Conductivity of Commercial Fibrous Carbons

#### 3.2.1. Experimental Measurements of Effective Thermal Conductivity

Thermal conductivity measurements were carried out for all commercial fibrous carbons, and the corresponding data are given in Table S7 of the Supplementary Material. As already seen in Figure S12, the thermal conductivity of a single graphitic crystallite is anisotropic, therefore the thermal conductivity of the carbon fibers in which the crystallites are oriented, and that of the final nonwovens in which the fibers have a preferential orientation, is anisotropic as well. It is also known that, in general, by increasing the heat treatment temperature, the crystallites grow, have fewer and fewer defects and sometimes even graphitization may occur, which increases the thermal conductivity. However, the nature of the precursors of the fibers, their preparation process and their thermal history directly influence the final organization of the carbon, its graphitic character or not, its purity and its general organization within the fibers [21]. Finally, differences in structure and high porosity of the carbon nonwovens are further reason for the dispersion of thermal conductivity values in this and many other studies [59,60,61].

**Figure 11:**
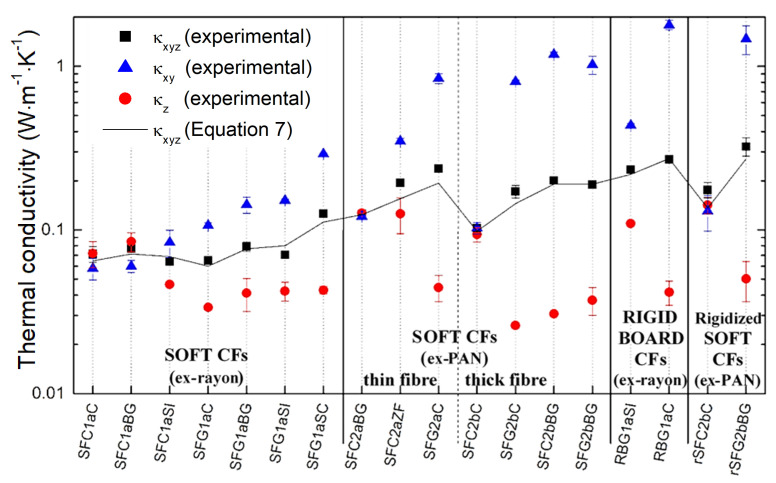
Thermal conductivity values of all commercial fibrous carbons measured by either isotropic mode (black squares—*κ_xyz_*) or anisotropic mode (blue triangles for the IP direction—*κ_xy_*, and red circles for the OP direction—*κ_z_*); the black solid line represents the calculated *κ_xyz_* from the separate measurements of *κ_xy_* and *κ_z_*, using Equation (7).

Figure 11 shows the effects of the nature of the carbon fibers and the nature of the corresponding fibrous materials on the values of thermal conductivity, obtained either by the isotropic or anisotropic mode of analysis. To confirm the validity of the results in the anisotropic mode, Equation (7) was used, and good agreement was observed between the measured and calculated thermal conductivity values. Indeed, as explained elsewhere [62,63], the IP and OP thermal conductivity obtained with a single transient plane source test in anisotropic mode can be correlated with the isotropic measurement using the following equation:

(7) $$\kappa_{\mathrm{xyz}}\;=\;\sqrt{\kappa_{\mathrm{xy}}\;\kappa_{z}},$$

where *κ_xyz_* is the overall thermal conductivity calculated from the experimental measurements of the thermal conductivity carried out on the one hand in the IP direction, leading to *κ_xy_*, and on the other hand in the OP direction, leading to *κ_z_*.

The validated measurements, obtained in the anisotropic analysis mode, can therefore be used to discuss the different heat transfer behaviors of the examined materials. As can be read in the literature [59,61], all measured values are 1 to 3 orders of magnitude lower than the axial conductivity of an individual carbon fiber. The highly porous structure of nonwovens, and thus the presence of an insulating gas phase occupying most of their volume, are therefore considered the main reasons for the dramatic decrease in measured thermal conductivity compared to that expected for a single carbon fiber. Experimental results of effective (i.e., measured from the contribution of both solid and gaseous phase) thermal conductivity, different in the OP and IP directions, confirm the anisotropic nature of commercial fibrous carbons. Indeed, the latter are generally much less conductive in the OP (*z*) direction than in the IP (*xy*) direction. The anisotropic thermal behavior is due to the preferential orientation of the fibers, which are much more numerous in the IP direction than in the OP direction, in good correlation with the µCT results already presented in Section 2.2 of the Supplementary Material.

In the case of the IP thermal conductivity (*κ_xy_*), differences in the results can be attributed to the conductive nature of the individual fibers, provided that all other felt formation parameters are similar. The influence of fiber conductivity is observed from the corresponding soft CFs, i.e., without or with graphitization, such as SFC1aC↔SFG1aC, SFC1aBG↔SFG1aBG, SFC1aSI↔SFG1aSI and SFC2bC↔SFG2bC. Produced only by increasing the final temperature, graphitized CFs show increased thermal conductivity and greater differences in values between the IP and OP directions. Exceptions, such as SFC2bBG, even though only carbonized at about 1200 °C (information from the manufacturer) showed a highly directional and anisotropic thermal conductivity similar to that of the corresponding graphitized SFG2bBG. The latter correlates well with the examined structural properties of SFC2bBG [21], which is considered the only carbonized CF with an improved turbostratic structure similar to that of the corresponding graphitized SFG2bBG. These advantageous characteristics are based on the precursors used in the manufacture of SFC2bBG, giving it a high carbon purity and improved structural order compared to the other carbonized CFs evaluated.

On the other hand, the thermal conductivity in the OP direction depends mainly on the thermal resistance between the soft or rigid contacts and the proportion of carbon fibers reoriented along the *z*-axis by the needle-punching process. Similar observations have been made elsewhere [59], showing that thermal conductivity is more affected in the OP direction (*κ_z_*) than in the IP direction (*κ_xy_*) by changes in the tortuosity of the felts and the reorientation of the fibers.

In the case of all carbonized CFs (except SFC2bBG and SFC2aZF), i.e., heat-treated at a temperature ≤1200 °C, we obtained close thermal conductivity values in both IP and OP directions compared to the graphitized ones. This observation is explained by the less conductive nature of the constituting carbon fibers compared to their graphitized counterparts. Although this may seem obvious, the difference in thermal conductivity in the IP and OP directions is smaller for highly needle-punched CFs, such as SFC2aZF, compared to other materials whose fibers are more oriented in the IP direction. These results correlate well with the results obtained by µCT analysis. A similar effect has been observed in previous studies [64,65,66], which confirmed the influence of needle-punching density on thermal conductivity by producing a series of CFs with different needle-punching densities. Therefore, the combined effects of the conductive nature and orientation of the carbon fibers influences the anisotropic heat transfer behavior. When studying commercial CFs from different suppliers, it is difficult to evaluate the effect of separate parameters due to the diversity of manufacturing techniques.

The soft Rayon-derived CFs evaluated are generally much less conductive in the IP direction than the soft PAN-derived CFs, when compared to CFs manufactured with similar final heat treatment. This may be due to the higher aspect ratio of the PAN-derived carbon fibers and their circular cross-section, but mainly to the graphitic structure, which results in higher thermal conductivity in the axial direction of the fibers compared to the Rayon-derived fibers. A similar comparison cannot be made with rigid boards and rigidized soft CFs, due to the effect of the binder (possibly introduced in different amounts) and to the different methods to prepare them.

The thermal conductivity of the rigid boards again demonstrates their anisotropic structure, due to the preferential orientation of the fibers and the inhomogeneous dispersion of the binder at the interconnections. A better thermal conductivity in the IP direction is observed due to a higher proportion of interconnected fibers connections, as in ex-Rayon materials. The observed results are thus in good agreement with former studies, suggesting the strong influence of fiber and binder continuity on the thermal conductivity of fibrous carbons [24,67].

**Figure 12:**
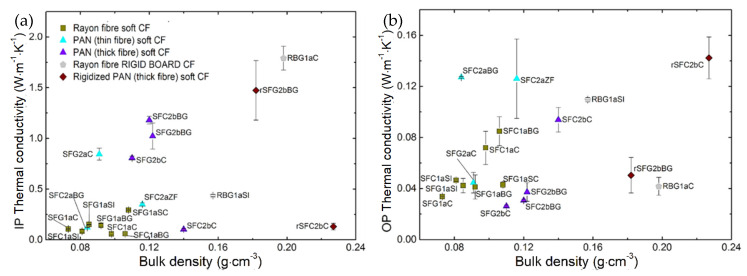
Thermal conductivity of all materials in: (**a**) IP direction and (**b**) OP direction, as a function of bulk density. The symbols in (**b**) have the same meaning as in (**a**).

Figure 12 shows the increase in the effective thermal conductivity of CFs with bulk density. Since the fiber orientation in all of the materials studied here is superior in IP than in the OP direction, it appears that the effective thermal conductivity is less influenced by the density variation in the IP direction than in the OP direction. A higher bulk density should result in higher carbon fiber content per unit volume and smaller pores between the fibers. Therefore, since the thermal conductivity of carbon fibers is much higher than that of air (at least 200 times higher), its contribution to the effective thermal conductivity increases with the felt density. The increase in bulk density can also be correlated with the increase in needle-punching density. Likewise, the felt becomes more compact, with more needle-punched fibers oriented in the OP direction, resulting in a further increase in thermal conductivity. In the case of rigid boards and rigidized soft CFs, the thermal resistance at the fiber-fiber contacts decreases as the binder concentration increases, which is correlated with the increase in fabric density. Again, clearer observations were prevented by significant differences in fiber/felt structure and differences in the final heat treatment.

#### 3.2.2. Effective Thermal Conductivity of CFs Subjected to Compression

In many applications, such as fuel cells or redox flow batteries [59,60,61,68,69], various fibrous carbons are used and habitually compressed between different composite layers. Moreover, the conditions imposed should further influence the effective thermal conductivity of the CFs by changing the contacts between the fibers and the porosity of the felts, i.e., the structure of felts.

**Figure 13:**
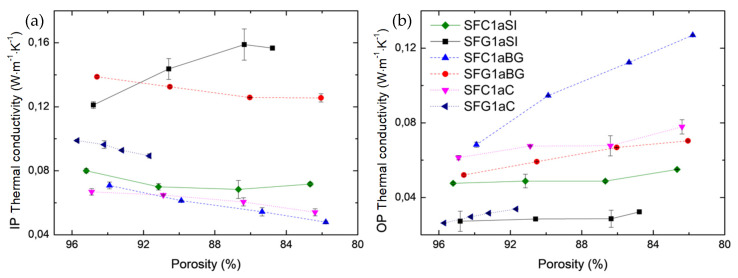
Thermal conductivity of Rayon-derived soft CFs as a function of porosity, at the initial material porosity and after each decrease (under load), in both directions: (**a**) in-plane and (**b**) out-of-plane. The symbols in (**b**) have the same meaning as in (**a**). The lines are just guides for the eye.

In order to analyze the influence of compression on the effective thermal conductivity, the experimental set-up described above was reused in a modified version. The measurements were carried out on samples from a single subgroup of the Rayon-derived soft CFs. This subgroup was chosen because it is the only containing three pairs of graphitized -non-graphitized CFs. The method used, described in Section 4.1 of the Supplementary Material, shows that effective thermal conductivity is strongly affected by the increase in compressive loading, as seen in Figure 13 and as detailed in Table S8.

According to our previous research on mechanical properties, the decrease in thickness, and therefore porosity, of materials under compression should not lead to changes in the morphology of fibers in terms of cross-section or length. Moreover, the observed linear change of the thermal conductivity, in both IP and OP directions, should only result from the change in the structure of the felts and the improvement of the contacts between the fibers, i.e., the decrease in thermal resistance at the contacts between the fibers. Deviations from the linear variation mentioned above are only observed for SFC1aSI and SFG1aSI. Thus, these deviations correlate well with the calculated elastic limit of these materials (Figure 6), which was very low and which always resulted in a change of less than 5% in the initial porosity of the materials under dynamic compression.

Figure 13 shows the thermal conductivity as a function of porosity, from initial porosity (without compression) to lower values (under load), in both IP and OP directions. Note that the axis of porosity in Figure 13 is reversed, i.e., the values decrease from left to right, to go in the direction of the increase in pressure applied to the felts. In all the CFs examined, the increased number and area of fiber contacts should lead to the observed increase in thermal conductivity in the OP direction. Again, the high initial thermal resistance at the contacts is assumed to be the main factor in the low heat transfer and temperature distributions in this direction [69,70]. It can be seen that the increase in thermal conductivity in the OP direction is strongly dependent on the type of material considered in the same range of porosity. Moreover, the improved carbon structure in SFC1aBG resulted in a more marked increase in thermal conductivity compared to other carbonized CFs (SFC1aSI or SFC1aC) in the examined porosity range. In addition, it can be seen that the thermal conductivity decreases in the IP direction while it increases in the perpendicular direction. Again, this trend is not observed or is altered in the case of SFC1aSI and SFG1aSI due to possible changes in the fiber morphology (e.g., length) as porosity decreases. In any case, the change in material thickness (porosity) has a greater influence on the effective thermal conductivity in the OP than in the IP direction.

Several analytical equations widely used in the literature were then tested to account for the experimental results shown in Figure 13. In particular, the following models were tested: the Maxwell model [59], an empirical model for the thermal conductivity of graphite felt [71], volume-average and fractal models [4,59,71,72,73,74] and the self-consistent model [4,75]. However, the desired thermal conductivity of the solid phase (carbon fiber) could not be precisely derived due to the poor fit of these models to the experimental results. The available models seem to be oversimplified and unsuitable for describing the thermal behavior of different complex nonwovens. All the models found in the literature that we have tested [4,59,71,72,73,74,75] were in fact only based on the effect of the porosity of the nonwovens, without taking into account other variable geometric parameters (such as angle distribution between fibers and/or fibers’ aspect ratio). Moreover, the analytical solutions neglect the great structural diversity of the commercial CFs, and they have only been developed from simplified structures of simulated fibrous materials. As already observed [59,73,74,76] and also acknowledged in this paper, the actual geometric and conductive properties of the various complex fibrous matrices make many volume-average and fractal models ineffective in the determination of commercial CFs’ thermal conductivity. Moreover, it is even more difficult to use the cited analytical models for studying CFs with identical or lower porosity but higher thermal conductivity, as shown by the present results.

## 4. Conclusions

In this study, the initial focus was on identifying the uniaxial compressive behavior, governed by different mechanisms, of various commercial fibrous carbon materials. It was seen that the elastic properties of most soft CFs are very complex, with an almost continuous non-linear evolution of stress at constant deformation rate, in contrast to the successive regions of linear compression, buckling and densification observed for rigid boards and rigidized CFs.

Due to the need to model the elastic behavior of soft CFs and the impossibility of determining a single value of elastic modulus with the commonly used Hooke’s law, the Mooney–Rivlin model was chosen and applied because of its mathematical simplicity. It allowed us to determine analytically the effective elastic modulus of soft CFs. In addition to the “dynamic” method, the compressive behavior was also studied by a little-known “quasi-static” method. The possibility of using two different test methods made it possible to compare the results of effective elastic modulus obtained under different test regimes. A good correlation was found between the values calculated from the stress-strain curves and from the QMA. However, the QMA method did not provide an overall elastic modulus of the materials, and it is only appropriate for determining the effective elastic modulus of soft CFs at a predefined strain. In addition, and for the first time, the QMA method was successfully used to study the damping loss factor of commercial CFs.

The effective thermal conductivity of the commercial CFs examined was also considered in the second part of the study, and was found to be much lower than that of the constituent carbon fibers. This observation is due to the very high porosity of the materials studied, but also to various structural defects in the fibers and their organization in the felts, implying high thermal contact resistances. These results also show that the thermal conductivity of most CFs is highly anisotropic, with higher values in the IP direction than in the OP direction. Therefore, a higher heat transfer in the plane of the felts is always to be expected, and the materials should be properly adapted to the conditions of use of their final application. In addition, the thermal conductivity of the commercial CFs tested change due to compression. Thus, in most cases, the thermal contact resistance decreases linearly with decreasing material thickness (porosity), and more in the OP than in the IP direction. Finally, the unsuccessful fitting of the results by different thermal conductivity models found in the literature requires further research to develop additional characterization tools adapted to this type of complex materials. To date, the results of effective thermal conductivity at different material thicknesses (porosities) are not available in the literature.

Through this detailed study, the structural and physical properties of fibrous carbons can be adjusted to develop materials with optimal performance in various applications.

## Figures and Tables

**Table 1:** Sorting of the CFs investigated here into groups, depending on their main characteristics. “Ave.” and “Stdv.” stand for average and standard deviation, respectively.

Sample Code	Result (MPa)	Bulk Density (g cm^−3^)	Overall Porosity (%)	Elastic Modulus *E* (MPa)	Yield Stress (MPa)
**Rigidized PAN (Thick Fibers) CFs**					
rSFG2bBG	Ave.	0.182	89.3	2.26	0.045
	Stdv.	0.010	—	0.28	0.001
rSFC2bC	Ave.	0.227	88.1	1.81	0.102
	Stdv.	0.014	—	0.46	0.041
**Rayon rigid boards**					
RBG1aSI	Ave.	0.157	89.6	13.48	0.171
	Stdv.	0.012	—	0.25	0.001
RBG1aC	Ave.	0.198	87.5	20.30	0.360
	Stdv.	0.000	—	0.76	0.002

**Table 2:** Mooney-Rivlin constants identified from fitting Equation (4) to the experimental data and values of the elastic modulus, E*, obtained by application of Equations (5) and (6).

Sample Code	Experimental Data Fit with Equation (4)			Elastic Modulus E*
	*C*_10_	*C*_01_	R^2^	(MPa)
**RAYON soft CFs**				
SFG1aBG	0.031	0.005	0.992	0.142
SFC1aBG	0.026	0.009	0.991	0.140
SFG1aSC	0.016	0.005	0.996	0.083
SFC1aC	0.008	0.010	0.994	0.074
SFG1aC	0.005	0.009	0.994	0.054
SFC1aSI	0.004	0.005	0.997	0.040
SFG1aSI	−0.009	0.012	0.999	0.012
**PAN (thin fibers) soft CFs**				
SFC2aZF	0.019	0.006	0.998	0.107
SFC2aBG	0.008	0.007	0.998	0.060
SFC2aC	0.013	0.001	0.998	0.057
**PAN (thick fibers) soft CFs**				
SFC2bBG	0.073	0.006	0.992	0.320
SFC2bC	0.030	0.016	0.996	0.190
SFG2bBG	0.049	-0.002	0.994	0.187

## Data Availability

Data is contained within the article.

## Supplementary Materials

The following are available online at https://www.mdpi.com/article/10.3390/ma14071796/s1; Table S1: Classification by groups of the fibrous carbons studied in this work, according to their main characteristics. “CFs” means “carbon felts”, Table S2: Property data available for all commercial fibrous carbon samples received and investigated here, as provided by the suppliers, Figure S1: Schematic representation of X-ray tomography apparatus, Table S3: Comparison of porosities calculated in [3] and deduced from the present μ-CT analysis, Figure S2: 3D rendering of the binarized data from μ-CT along the IP (*xy* view) and OP (*z* view) directions, and 3D reconstructions of the rigid board derived from Rayon: RBG1aC; additional table showing the statistical output of 3D space orientation analysis—the mean orientation tensor, Figure S3: Same as in Figure S2 but for Rayon soft CFs: (a) SFG1aBG and (b) SFG1aSC, Figure S4: Same as in Figure S2 but for PAN-derived soft CFs: (a) SFC2aZF (thin fiber) and (b) SFG2bC (thick fiber), Section 3: Mechanical Compression Tests of Commercial Fibrous Carbons, Figure S5: Typical stress-strain curve of brittle (solid line) and (hyper)elastic (dashed line) fibrous carbons subjected to compression, Figure S6: Measurement of the mechanical properties of fibrous carbons under compression using an Instron 5944 universal testing machine. The insert shows the compression platens on either side of a specimen just prior to its compression, Table S4: Compression stresses at three different strains, and structural characteristics of soft and rigidized CFs. “Ave.” stands for average, Figure S7: Experimental stress-strain curves (black lines) and fit by the Mooney-Rivlin equation (Equation (4)) (red lines) for soft CFs derived from Rayon under uniaxial compression; Figure S8: Same as Figure S7 but for: (a) PAN-derived (thick fiber) and (b) PAN-derived (thin fiber) soft CFs; Table S5: Elastic modulus, *E*, calculated by applying Equation (1) to the linear elastic part of the stress-strain curves of PAN-derived (thin fibers) soft CFs, Figure S9: Scheme (left) and real representation (right) of quasi-static mechanical analyzer (QMA) and installed soft CF sample, with *x*-, *y*-, *z*-directions, Figure S10: Measurements of stiffness (blue and red lines stand for real and imaginary parts of the stiffness) as a function of strain (called “compression rate” on the graph), Table S6: Elastic modulus and damping loss factor of the soft CFs obtained by QMA measurements, with additional information on materials: fiber diameter, bulk density, porosity, and strain used. “Ave.” and “Stdv.” stand for average and standard deviation, respectively, Figure S12: Three levels of anisotropy observed in carbon nonwovens accounting for the anisotropic nature of their thermal conductivity (after [5]), Figure S13: Hot Disk TPS 2500 device with details of the measurement probe and the installation of a CF sample for testing, Table S7: Thermal conductivity obtained in isotropic and anisotropic analysis mode, with additional information of fiber diameter, bulk density and porosity. “Ave.” and “Stdv.” stand for average and standard deviation, respectively, Figure S14: Experimental testing setup: 1—Hot Disk sensor (connected to the Hot Disk TPS 2500 device) installed between the upper and lower samples, thus, fixed by support made of insulating material and adjustable in height; 2—Compression platens of the Instron device; 3—Draught-free enclosure; 4—Schematic presentation of the CF sample set for testing., Table S8: Thermal conductivity of Rayon-derived softs CFs subjected to compression, obtained in isotropic and anisotropic mode, with additional information on modified bulk density and porosity of the sample.
