# Supplementary material for: Mechanical and Thermal Behavior of Fibrous Carbon Materials
Source: Materials (Basel). 2021 Apr 5;14(7):1796. doi: 10.3390/ma14071796 (PMC8038575; doi:10.3390/ma14071796)
Supplement: Supplementary file 1 [file materials-14-01796-s001.pdf]

materials

MDPI

Supplementary Material

Mechanical and Thermal Behaviour of Fibrous Carbon Materials

Blagoj Karakashov [1], M'Barek Taghite [2], Richard Kouitat [2], Vanessa Fierro [1] and Alain Celzard [1]*

[1] Institut Jean Lamour (IJL), Université de Lorraine, CNRS, F-88000 Epinal, France
[2] Institut Jean Lamour (IJL), Université de Lorraine, CNRS, F-54000 Nancy, France
* Correspondence: alain.celzard@univ-lorraine.fr; Tel.: + 33 372 74 96 14.

Citation: Karakashov, B.; Taghite, B.; Kouitat, R.; Fierro, V.; Celzard, A. Mechanical and Thermal Behavior of Fibrous Carbon Materials. *Materials* **2021**, *14*, 1796. https://doi.org/10.3390/ma14071796

Academic Editor: Miguel Jorge

Received: 24 February 2021
Accepted: 28 March 2021
Published: 5 April 2021

Publisher's Note: MDPI stays neutral with regard to jurisdictional claims in published maps and institutional affiliations.

Copyright: © 2021 by the authors. Licensee MDPI, Basel, Switzerland. This article is an open access article distributed under the terms and conditions of the Creative Commons Attribution (CC BY) license (http://creativecommons.org/licenses/by/4.0/).

Section 1. Classification of Commercial Fibrous Carbons and Data Available from the Suppliers

Table S1. Classification by groups of the fibrous carbons studied in this work, according to their main characteristics. "CFs" means "carbon felts".

Final Production Heat Treatment	Precursor	Fibre Diameter	Commercial Name:	Used Sample Code:
**Needle-punched non-woven Soft CFs**				
Carbonised			Carbon (Rayon) felt CeraMaterials	SFC1aC
Graphitised			Graphite (Rayon) felt CeraMaterials	SFG1aC
Carbonised	Rayon	9–12 µm	RSF1 Beijing Great Wall Co.	SFC1aBG
Graphitised			RSF2 Beijing Great Wall Co.	SFG1aBG
Carbonised			SIGRATHERM® KFA5	SFC1aSI
Graphitised			SIGRATHERM® GFA10	SFG1aSI
Graphitised			GF2 Schunk	SFG1aSC
Carbonised			PX 35 ZOLTEK™	SFC2aZF
Graphitised		9–10 µm	GFE-1 CeraMaterials	SFG2aC
Carbonised			BESF Beijing Great Wall Co.	SFC2aBG
Carbonised	PAN		Carbon (PAN) felt CeraMaterials	SFC2bC
Graphitised		17–20 µm	Graphite (PAN) felt CeraMaterials	SFG2bC*
Carbonised			PSF1 Beijing Great Wall Co.	SFC2bBG
Graphitised			PSF2 Beijing Great Wall Co.	SFG2bBG
**Chemically rigidized needle-punched non-woven Soft CFs**				
Graphitised	PAN	17–20 µm	PRF3 Beijing Great Wall Co.	rSFG2bBG
Carbonised			Carbon Board CeraMaterials	rSFC2bC
**Chemically bonded Rigid Boards**				
Carbonised	Rayon	9–12 µm	SIGRATHERM® MFA	RBG1aSI
Graphitised			Graphite Board CeraMaterials	RBG1aC

* This material was not considered for mechanical properties as the original carbon felt was received folded from the supplier, and we were unable to cut a correct sample with the large dimensions required for mechanical testing, either by the non-destructive or destructive method.

Table S2. Property data available for all commercial fibrous carbon samples received and investigated here, as provided by the suppliers.

SUPPLIER	SGL Carbon Group			CeraMaterials							Schunk	Beijing Graphite Great Wall						ZOLTEK
**USED CODE:**	**SFC1aSI**	**SFG1aSI**	**RBG1aSI**	**SFC2bC**	**SFC1aC**	**SFG1aC**	**SFG2bC**	**SFG2aC**	**rSFC2bC**	**RBG1aC**	**SFG1aSC**	**SFC1aBG**	**SFC2bBG**	**SFG1aBG**	**SFG2bBG**	**SFC2aBG**	**rSFG2bBG**	**SFC2aZF**
**Carbon fibre precursor**	/	Rayon	/	PAN	Rayon	Rayon	PAN	PAN	PAN	Rayon	Rayon	Rayon	PAN	Rayon	PAN	PAN	PAN	PAN
**Heat treatment (°C) (inert atmosphere)**	1000	2000	2000	1200	1200	2000	2000	/	/	2000	/	1200	1200	2200-2400	2200-2400	/	/	/
**Thickness (cm)**	0.65	1.15	/	0.635 - 5.08	0.635 - 5.09	0.635 - 5.11	0.635 - 5.10	0.1 - 1.5 (±0.35)	/	/	1	/	/	/	/	/	/	1.27
**Fibre diameter (μm)**	/	10 - 12	/	/	/	/	/	/	/	/	/	/	/	/	/	/	/	12.5
**Carbon content (%)**	/	/	/	≥ 97	≥ 97	≥ 99	≥ 99	99.8	/	/	/	/	/	/	/	/	/	95
**Ash content (% ') or (ppm *)**	1.7 '	≤ 400 *	1000*	≤1 '	≤1 '	≤ 0.5 '	≤ 0.5 '	/	/	/	≤ 300 *	/	/	/	/	/	/	/
**Weight per unit area (g/m²)**	540	1000	/	/	/	/	/	/	/	/	/	/	/	/	/	/	/	1700
**Bulk density (g/cm³)**	/	/	0.2	0.12	0.15	0.085	0.12	0.09 - 0.15	/	/	/	/	/	/	/	/	/	0.102
**Thermal conductivity (at 23°C) (W /m.K)**	/	/	0.22	0.22	0.21	0.29	0.35	/	/	/	/	/	/	/	/	/	/	/

0

Section 2. Tomography studies of the structure of fibrous carbons

Section 2.1. Materials and Methods

X-ray tomography is a non-destructive imaging method that can be used to characterise the microstructure of porous media [1]. By rotating a sample 180° in an X-ray beam, a radiographic projection is made at each degree, and the series of projections is then used to construct 2D sections. The X-ray beam is attenuated by the presence of material and these intensity variations, related to the differences of density of the material, are measured by the detector array. The attenuation of the beam is usually represented by 2D grey scale pixels. Figure S1 shows a schematic diagram of an X-ray tomography device.

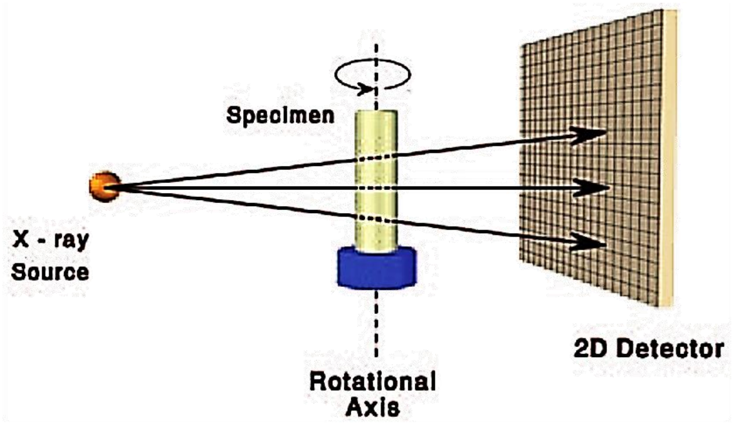

Figure S1. Schematic representation of an X-ray tomography apparatus.

Based on the acquired 2D greyscale images and their further processing, a reconstruction of the original 3D microstructure can be provided. A 3D material model obtained by X-ray tomography can be used to present the structural properties of the fibrous material at the microscopic scale [2].

To generate the 3D images of five different fibrous carbons, X-ray micro-computed tomography (μ-CT) studies were carried out. X-rays were generated at 60 kV to form a beam current of 150 μA. The samples for μ-CT had diameters of 4–6 mm and heights (thicknesses) imposed by their manufacturing process. Finally, a set of reconstructed 3D cross-sectional images was obtained using VGStudio software (version 3.22.30436). The pixels of the reconstructed cross-section images in the xy-plane (in-plane direction) were stacked along the z-axis (out-of-plane direction) to build the D image of the cylindrical object. The voxel size, 4 μm in side, was recorded so that the physical size could be identified. Prior to performing the μ-CT scans, efforts were made to minimise the possible effects of cutting the sample on its structure, as well as to correctly align the prepared sample with respect to the x, y and z reference axes and those of the tomographic device.

Based on the obtained 3D reconstruction of the samples, precise quantifications of the morphology, using the finite volume method (used for the discretization of numerical simulation of various types), were carried out, and complement the studies carried out by scanning electron microscopy (SEM), i.e., in 2D, which has already been published [3]. Since SEM analyses are limited to the outer parts of the samples, μ-CT has proved that it can represent the entire material, allowing a highly reproducible 3D virtual reconstruction of the fibre networks [4]. Here, two soft CFs derived from Rayon, two soft CFs derived from PAN and one rigid board were imaged due to the differences in structure and porosity highlighted by the previous study. These differences may be the result of different manufacturing processes, specific to each supplier and to each defined type of commercial fibrous carbon. Although the scans obtained were performed at a resolution limited by the sample size, the technique nevertheless provided valuable information on the volume and orientation of the fibres in the carbon nonwovens.

Section 2.2. Structural Analysis

The imaging results are shown in Figure S2 to S4 below, which are considered as 3D photorealistic renderings of the materials seen from above (xy plane = IP direction) and seen on the edge (z axis = OP direction). For each material, an additional table is given in the figures with the statistical output of the 3D space orientation analysis, i.e., the mean orientation tensor. Thus, the 3D spatial orientation analyses were estimated at the microstructural level using the fibre analysis software module (in VGStudio MAX–Volume Graphics - version 3.22.30436). In order to obtain accurate analysis results, a specific simulation mesh (discrete geometric and topological cells (subdivisions) from continuous geometric spaces) was directly imported into the software, allowing the mean orientation tensor (representing the averaged fibre orientation) to be calculated for each cell of the mesh.

In addition to the averaged fiber orientation, the analysis explained above also provides information on the volume fraction of fibres, derived from the greyscale analysis. Table S3 below shows the porosity of the fibrous carbons, as previously defined and measured for the same materials [3]. More importantly, Table S3 allows comparison of the porosity and the pore volume fraction (pore volume fraction = 100—scanned fibre volume fraction) of a cylindrical volume defined in the field-of-view of the µ-CT analysis. It turns out that the estimates of the pore volume fraction obtained by calculation from the 2D slices (greyscale analysis) correlate well with the calculated overall porosity. Larger differences were only observed for SFG1aSC and SFG2bC. In both cases, the differences could be due to the cutting of very small cylindrical samples and thus to a low number of needle-punched fibres to maintain the integrity of the initial material, resulting in a slight increase in sample volume. The calculated value should also be handled with caution as the volume fraction of fibres is calculated based on grey level values, and areas between neighbouring carbon fibres can be misinterpreted as areas where fibres are present. Therefore, the overall pore volume fraction may be underestimated, as in the case of RBG1aC, which has been chemically consolidated.

Table S3. Comparison of porosities calculated in [3] and deduced from the present µ-CT analysis.

Sample Code	Overall Porosity Fraction (%)	Imaged Porosity Fraction (%)
	**Rayon soft CFs**	
SFG1aBG	94.6	94.42
SFG1aSC	92.7	96.45
	**PAN (thin fibres) soft CFs**	
SFC2aZF	93.4	93.53
	**PAN (thick fibres) soft CFs**	
SFG2bC	93.8	96.98
	**Rayon rigid board**	
RBG1aC	87.5	86.04

All the soft CFs examined showed a clearly needle-punched pattern, but with visibly different needle-punching densities, in accordance with former SEM studies. µ-CT also provides quantitative information on the orientations of carbon fibres. The fibres in soft CFs are mainly randomly and homogeneously oriented in the x-y plane, with the exception of SFG2bC (see Figure S4b). This is due to the use of a highly directional layering technique during the prefabrication of SFG2bC prior to the needle-punching process. In addition, the differences in fibre orientation along the z-axis are the result of the needle-punching process, which creates different fractions of fibre entanglements in the OP direction. While Rayon-derived soft CFs and SFG2bC (a PAN-derived (thick fibres) soft CF) present a lower fibre orientation in the OP direction compared to the IP direction, SFC2aZF (a PAN-derived (thin fibres) soft CF) is much more influenced by the barbed needles, as observed in Figure S4a. The increase in needle-punching density decreased the

orientation of the fibres in the IP direction by reorienting more fibres in the OP direction, resulting in an identical distribution of fibres in each of the *x*-,*y*- and *z*-axes.

The following three figures also highlight the significant structural differences between the rigid board RBG1aC and the soft CFs. The manufacturing technique used in the production of RBG1aC rigid board results in an almost transversely isotropic structure. However, RBG1aC has many fibre clusters and bundles due to the chemical consolidation process, which are not present in the needle-punched CFs. Therefore, it can be assumed that RBG1aC (see Figure S2) is manufactured by overspreading carbon fibres in the IP direction. However, the use of an organic binder and the observed variation in the length of fibres (considered as chopped or recycled carbon fibres of different lengths) ultimately results in a slightly modified felt morphology and an increased orientation of the fibres in the OP direction.

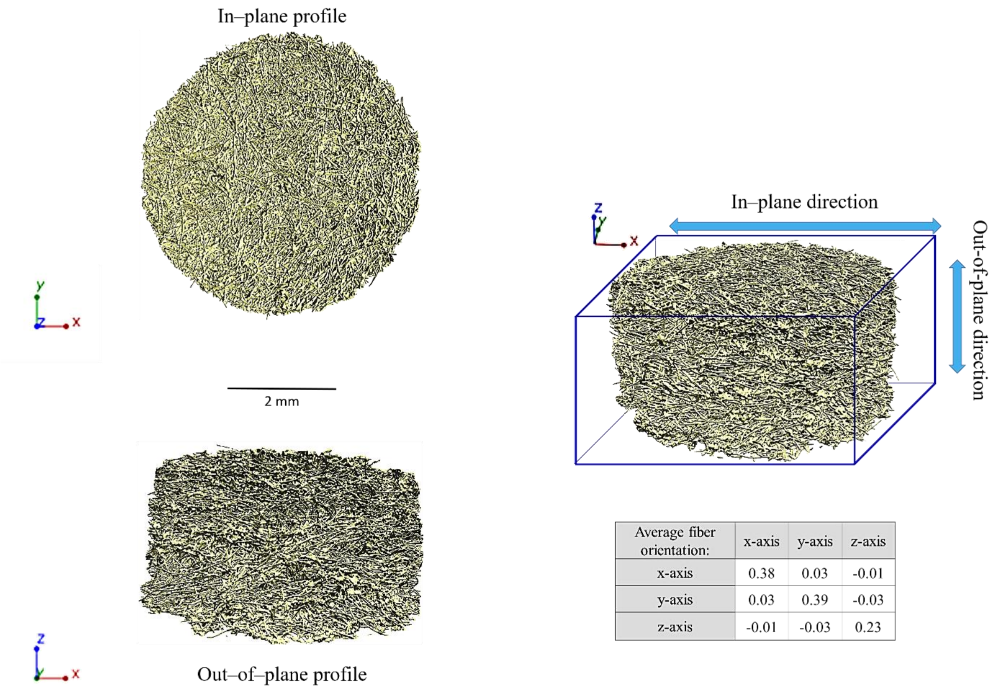

Average fiber orientation:	x-axis	y-axis	z-axis
x-axis	0.38	0.03	-0.01
y-axis	0.03	0.39	-0.03
z-axis	-0.01	-0.03	0.23

Figure S2. 3D rendering of the binarised data from μ-CT along the IP (*xy* view) and OP (*z* view) directions, and 3D reconstructions of the rigid board derived from Rayon: RBG1aC; additional table showing the statistical output of 3D space orientation analysis - the mean orientation tensor.

(a)

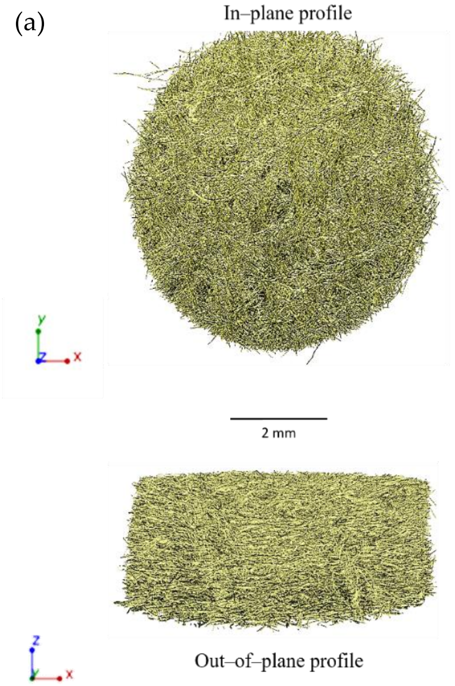

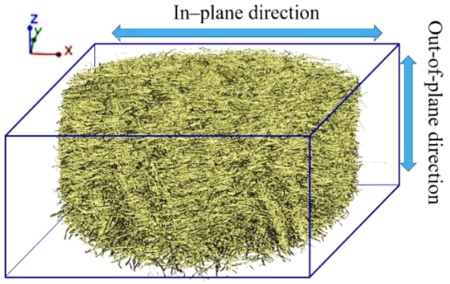

Average fiber orientation	x-axis	y-axis	z-axis
x-axis	0.37	0.01	0.00
y-axis	0.01	0.46	-0.05
z-axis	0.00	-0.05	0.17

(b)

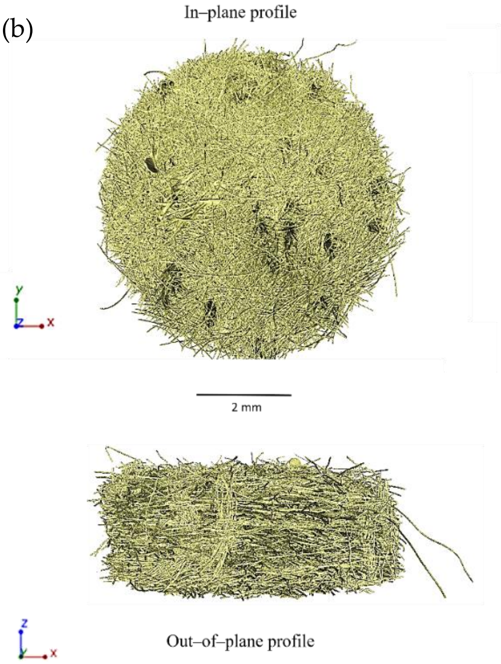

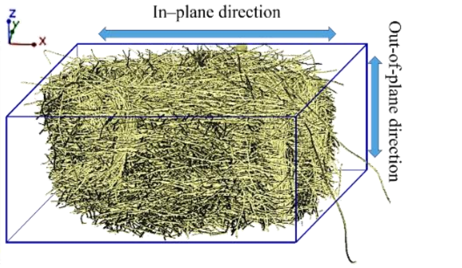

Average fiber orientation	x-axis	y-axis	z-axis
x-axis	0.43	0.02	-0.02
y-axis	0.02	0.40	-0.01
z-axis	-0.02	-0.01	0.17

Figure S3. 3D rendering of the binarised data from μ-CT along the IP (*xy* view) and OP (*z* view) directions, and 3D reconstructions of the rigid board derived for Rayon-derived soft CFs: (**a**) SFG1aBG; and (**b**) SFG1aSC; additional tables showing the statistical output of 3D space orientation analysis - the mean orientation tensor.

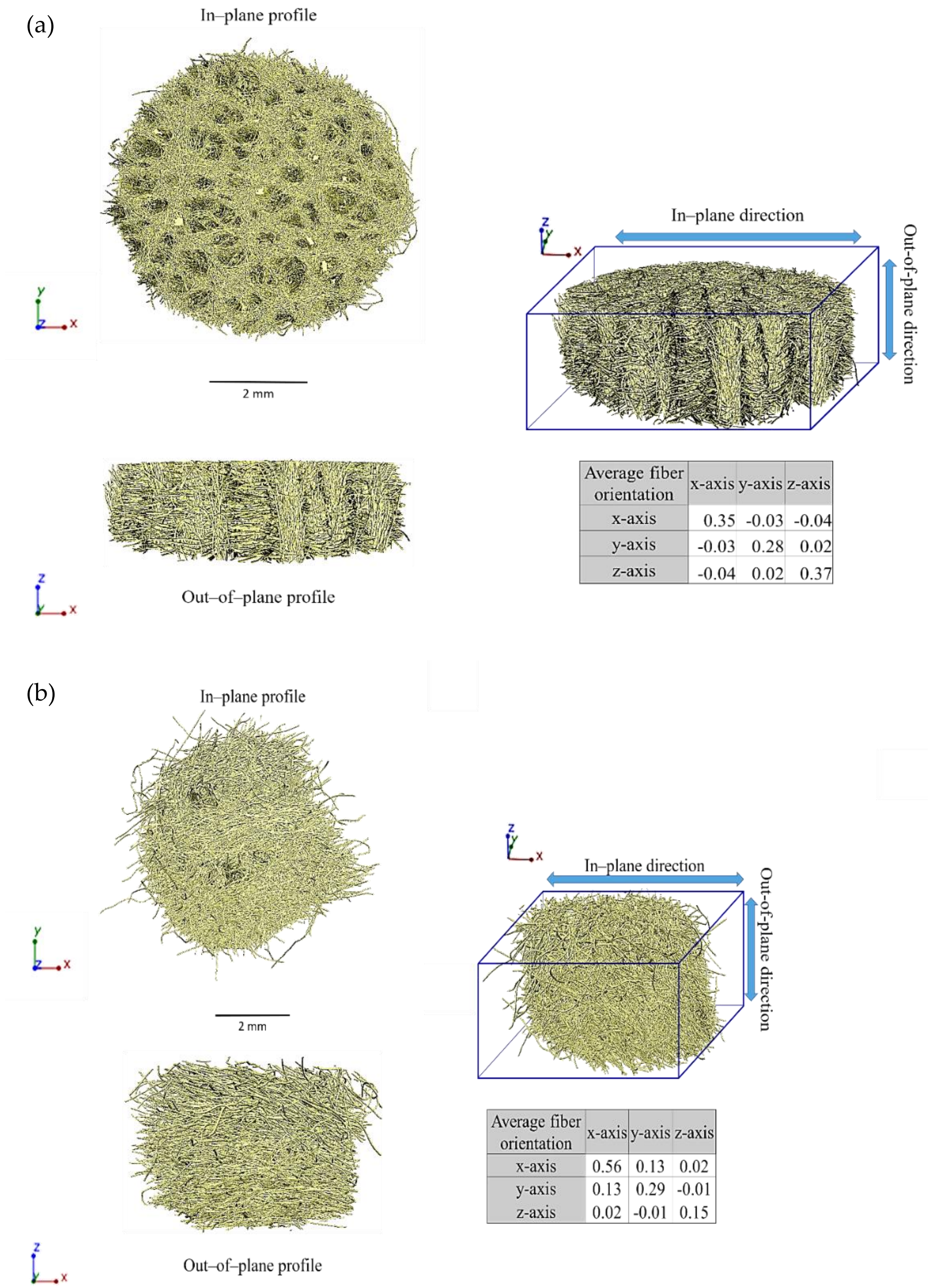

(a)

Average fiber orientation	x-axis	y-axis	z-axis
x-axis	0.35	-0.03	-0.04
y-axis	-0.03	0.28	0.02
z-axis	-0.04	0.02	0.37

(b)

Average fiber orientation	x-axis	y-axis	z-axis
x-axis	0.56	0.13	0.02
y-axis	0.13	0.29	-0.01
z-axis	0.02	-0.01	0.15

Figure S4. 3D rendering of the binarised data from μ-CT along the IP (*xy* view) and OP (*z* view) directions, and 3D reconstructions of the rigid board derived for PAN-derived soft CFs: (**a**) SFC2aZF (thin fibres); and (**b**) SFG2bC (thick fibres); additional tables showing the statistical output of 3D space orientation analysis - the mean orientation tensor.

Section 3. Mechanical Compression Tests of Commercial Fibrous Carbons

Section 3.1. Investigation by a Conventional Dynamic Compression Method

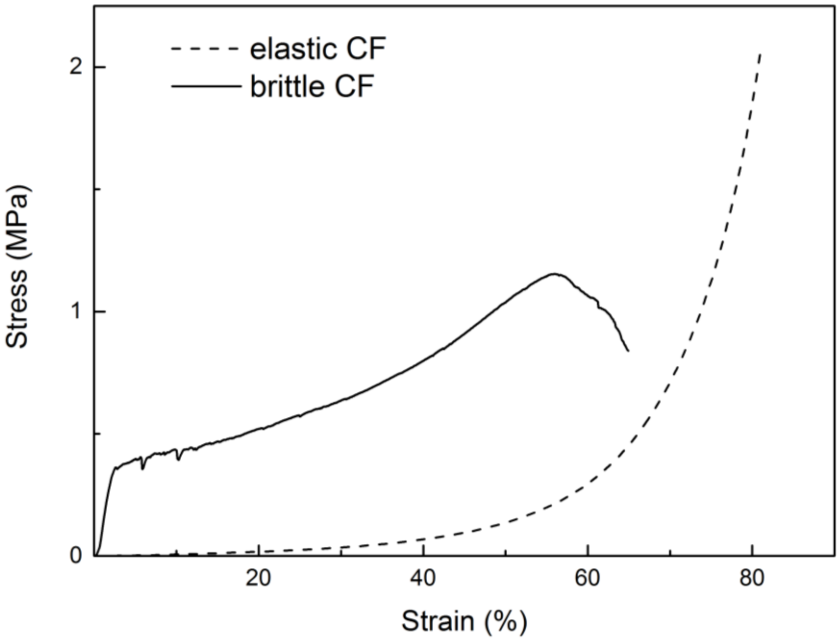

Figure S5. Typical stress-strain curve of brittle (solid line) and (hyper)elastic (dashed line) fibrous carbons subjected to compression.

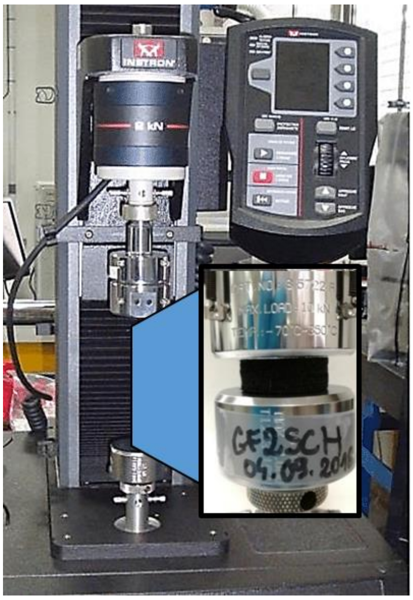

Figure S6. Measurement of the mechanical properties of fibrous carbons under compression using an Instron 5944 universal testing machine (INSTRON, 825 University Ave Norwood, MA, 02062-2643, USA). The insert shows the compression platens on either side of a specimen just prior to its compression.

Table 4. Compression stresses at three different strains, and structural characteristics of soft and rigidized CFs. "Ave." stands for average.

Sample Code	Fibre Diameter	Bulk Density	Overall Porosity	Stress [MPa]		
	Ave. (μm)	(g cm^{-3})	(%)	at 10 % Strain	at 30 % Strain	at 50 % Strain
			Rayon soft CFs			
**SFG1aSC**	10.30	0.108	92.7	0.006	0.034	0.137
**SFC1aBG**	11.98	0.106	93.9	0.004	0.041	0.193
**SFG1aBG**	12.57	0.092	94.6	0.003	0.033	0.178
**SFC1aC**	12.02	0.098	94.8	0.004	0.027	0.128
**SFG1aSI**	9.70	0.085	94.8	0.003	0.022	0.080
**SFC1aSI**	9.59	0.081	95.2	0.003	0.020	0.078
**SFG1aC**	10.70	0.073	95.7	0.001	0.015	0.091
			PAN (thick fibres) soft CFs			
**SFC2bC**	18.69	0.140	92.5	0.006	0.090	0.381
**SFC2bBG**	18.53	0.120	93.2	0.006	0.130	0.527
**SFG2bBG**	17.98	0.122	93.3	0.004	0.058	0.205
			PAN (thin fibres) soft CFs			
**SFC2aZF**	9.05	0.116	93.4	0.028	0.087	0.214
**SFC2aBG**	10.56	0.084	95.3	0.013	0.057	0.143
**SFG2aC**	9.06	0.091	95.0	0.009	0.041	0.091
			Rigidized PAN (thick fibres) CFs			
**rSFC2bC**	19.60	0.227	88.1	0.171	0.552	/
**rSFG2bBG**	17.00	0.182	89.3	0.200	0.565	/

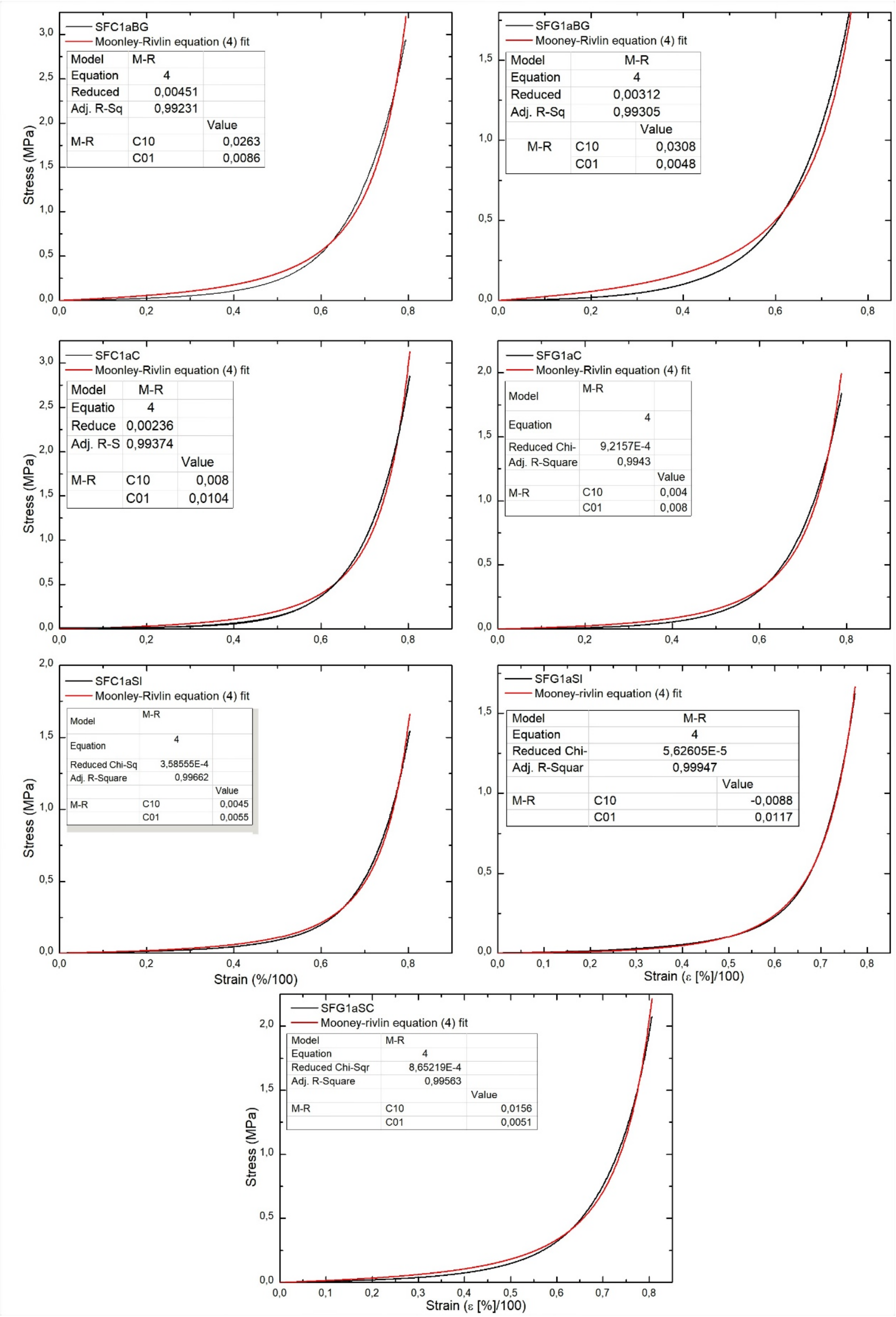

Figure S7. Experimental stress-strain curves (black lines) and fit by the Mooney-Rivlin equation (Equation (4)) (red lines) for soft CFs derived from Rayon under uniaxial compression.

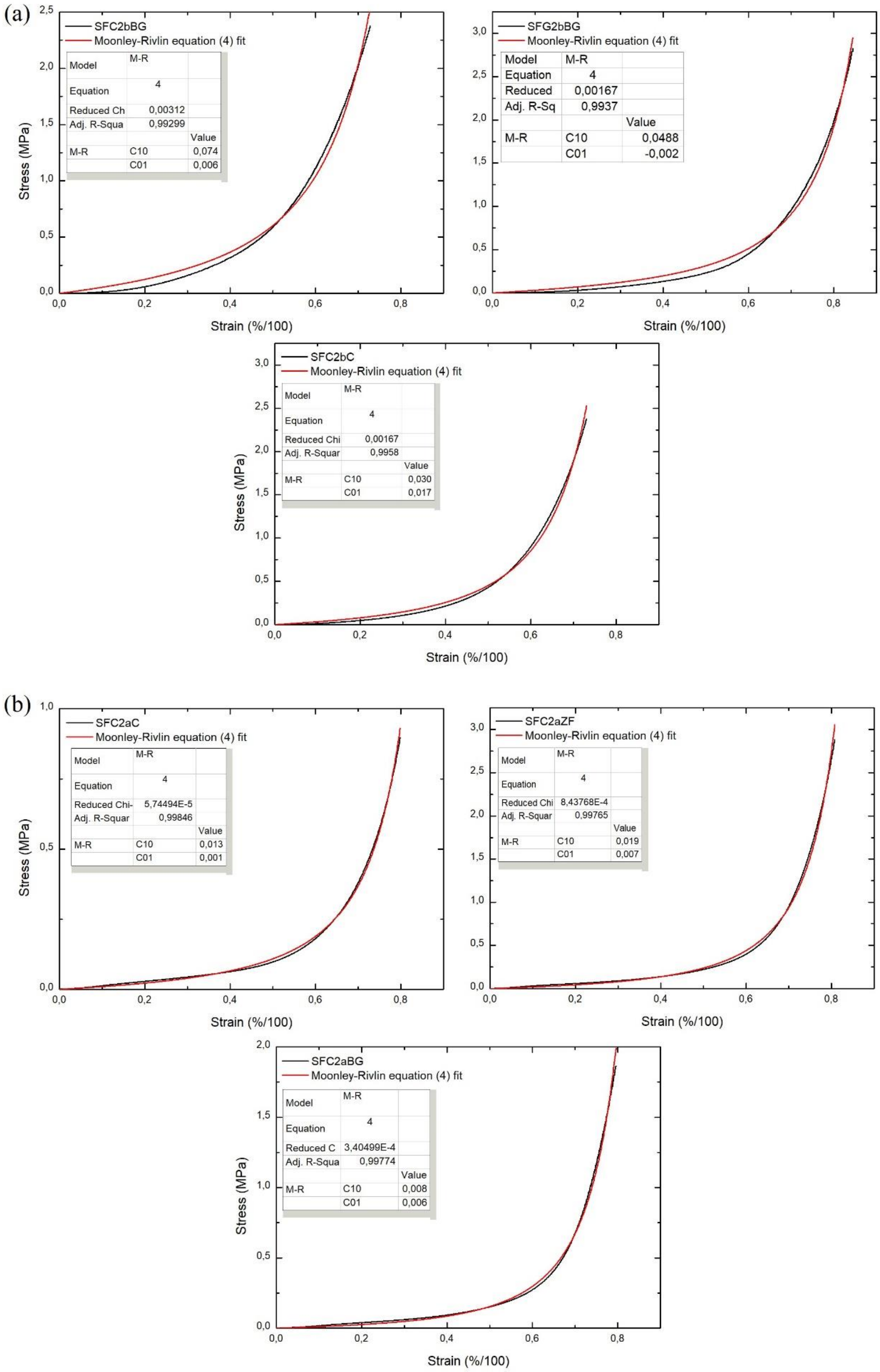

Figure S8. Experimental stress-strain curves (black lines) and fit by the Mooney-Rivlin equation (Equation (4)) (red lines from: a) PAN (thick fibres); and b) PAN (thin fibres) under uniaxial compression.

Table S5. Elastic modulus, *E*, calculated by applying Equation (1) to the linear elastic part of the stress-strain curves of PAN-derived (thin fibres) soft CFs.

Sample Code:	Elastic Modulus *E* [MPa]	Yield Stress [MPa]
	PAN (Thin Fibres) Soft CFs	
**SFC2aZF**	0.4	0.030
**SFC2aBG**	0.25	0.032
**SFG2aC**	0.17	0.019

Section 3.2. Investigation by a Non-destructive Method, the Quasi-static Mechanic Analysis (QMA)

The analysis procedure consists of placing the disc-shaped sample sandwiched between two rigid platens. The lower platen is excited axially by an electrodynamic shaker while the upper plate is fixed (see the scheme in Figure S9 (a)). The surfaces of the platens are coated with sandpaper to prevent the sample from slipping (see photo in Figure S9 (b)). A shaker with a pseudo-random noise with an unchanged frequency of 20 Hz is used to excite the lower platen, which is connected to an accelerometer, and a force transducer comes into contact with the upper platen. The accelerometer measures the acceleration of the lower platen, while the transducer measures the reaction force, provided by the sample, on the upper platen.

To ensure the linearity of the behaviour of porous materials, an ideal compression deformation must be determined. When testing materials of unknown stiffness, it is difficult to predict the ideal strain. In general, the measurements of stiffness versus strain are made to help identify the appropriate strain to be used so that the measured elastic properties do not depend on the experimental conditions. For this purpose, soft CFs have been tested in a strain range from 0.5 to 9.5 %, which are the limits predefined by the software to avoid damage to the test equipment. Figure S10 shows the different zones that should ideally be found during the tests, expressed as the real part of the stiffness versus the compression strain (blue curve). When a plateau is observed, it is recommended to select a strain in this region.

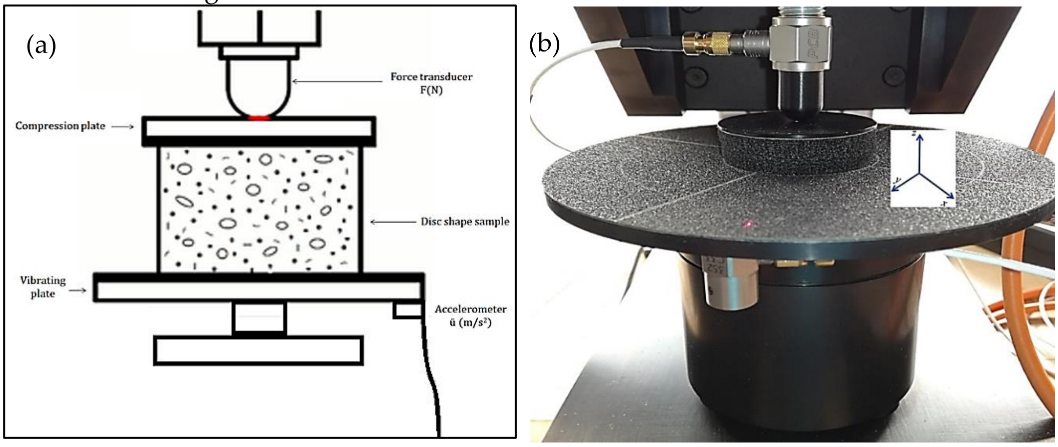

Figure S9. Scheme (a) and real representation (b) of the quasi-static mechanical analyser (QMA) and installed soft CF sample, with *x*-, *y*-, *z*-directions.

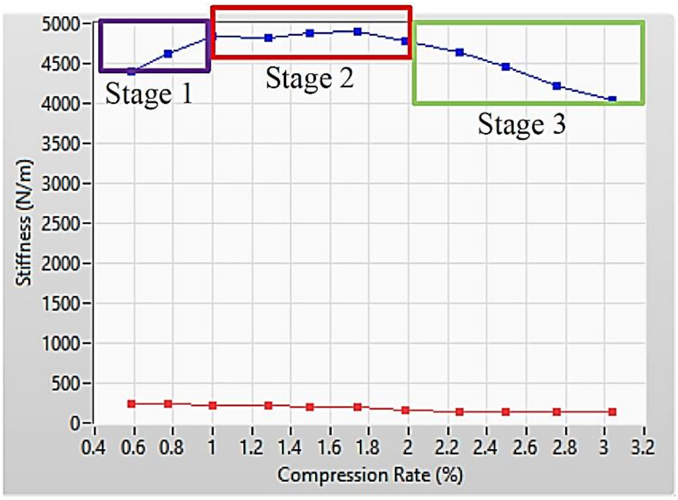

Figure S10. Measurements of stiffness (blue and red lines stand for real and imaginary parts of the stiffness) as a function of strain (called "compression rate" on the graph).

Table S6. Elastic modulus and damping loss factor of the soft CFs obtained by QMA measurements, with additional information on materials: fibre diameter, bulk density, porosity, and strain used. "Ave." and "Stdv." stand for average and standard deviation, respectively.

Sample code:	Fibre Diameter Ave. (μm)	Bulk Density ($g\ cm^{-3}$)	Overall Porosity (%)	Compression Strain (%)	Elastic Modulus (E) (MPa)	Stdv. (MPa)	Loss Factor (η)	Stdv.
				Rayon Soft CFs				
SFG1aC	10.7	0.073	95.7	1–3	0.036	0.009	0.181	0.007
SFC1aSI	9.59	0.081	95.2	1-3	0.053	0.016	0.354	0.014
SFC1aC	12.02	0.098	94.8	1-3	0.157	0.055	0.324	0.043
SFG1aSI	9.7	0.085	94.8	1-3	0.058	0.010	0.291	0.008
SFG1aBG	12.57	0.092	94.6	1-3	0.047	0.022	0.226	0.016
SFC1aBG	11.98	0.106	93.9	1-3	0.027	0.007	0.436	0.024
SFG1aSC	10.3	0.108	92.7	1-3	0.122	0.045	0.408	0.030
				PAN (thick fibres) soft CFs				
SFG2bBG	17.98	0.122	93.3	2-3	0.046	0.028	0.327	0.002
SFC2bBG	18.53	0.12	93.2	2-3	0.082	0.043	0.274	0.018
SFC2bC	18.69	0.14	92.5	2-3	0.216	0.079	0.316	0.048
				PAN (thin fibres) soft CFs				
SFC2aBG	10.56	0.084	95.3	1-2	0.237	0.036	0.366	0.035
SFG2aC	9.06	0.091	95	1-2	0.213	0.082	0.430	0.047
SFC2aZF	9.05	0.116	93.4	1-2	0.422	0.105	0.392	0.004

Section 3.3. Comparison of Destructive and Non-destructive Methods

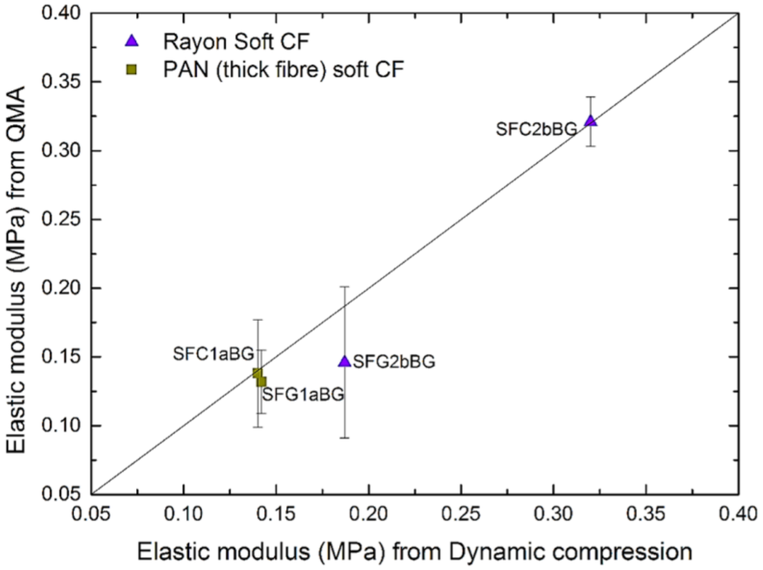

Figure S11. Relations between the elastic moduli measured by QMA (at 5–6% strain) and by the dynamic compression method, for soft CFs provided by the same supplier.

Section 4. Thermal Conductivity of Commercial Fibrous Carbons

Section 4.1. Anisotropic Properties of Fibrous Carbon Materials

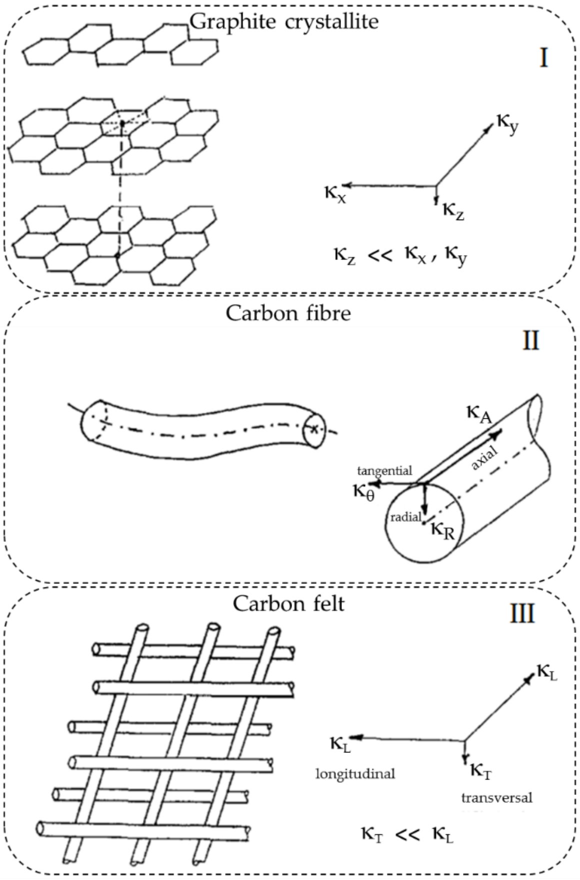

Figure S12. Three levels of anisotropy observed in carbon nonwovens accounting for the anisotropic nature of their thermal conductivity (Reproduced with permission from [5], published by Revue Générale de Thermique, Elsevier, (1997)).

Section 4.2. Measurement of the Effective Thermal Conductivity of Fibrous Carbons

The thermal conductivity of all carbon materials with different porosities was measured with a Hot Disk TPS 2500 device (Hot Disk AB, Sven Hultins gata 9A 412 88 Göteborg, Sweden) under fixed conditions of temperature (20 °C) and relative humidity (50%). A flat double spiral of nickel coated with Kapton® (the choice of diameter was determined by the experimental conditions), acting both as a heater and a temperature sensor, was sandwiched between two identical samples with a smooth surface and dimensions sufficient to consider the samples as semi-infinite media (see Figure S13). By imposing a heat pulse at a controlled power on the materials, the temperature response was recorded as a function of time. In general, with such a system, highly porous and poorly conducting materials raise the temperature significantly, even at low heating power. As a result, the stabilisation time (time between measurements on the same sample) was always greater than 30 minutes. The thermal conductivity was calculated using the Hot Disk software version 7.2.8, upgraded with an additional lab-made code kindly provided by I_2M–Bordeaux (UMR CNRS 5295) to improve the accuracy of the measurements by taking into account the contact resistance between the sensor and the material under study.

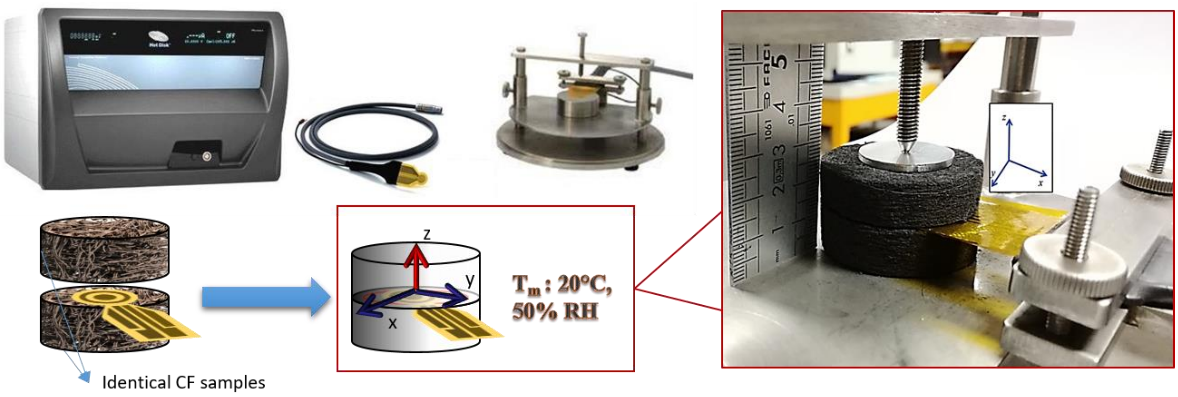

Figure S13. Hot Disk® TPS 2500 device with details of the measurement probe and the installation of a CF sample for testing.Section 4.3. Measurement of the effective thermal conductivity of CFs subjected to compression.

Table S7. Thermal conductivity obtained in isotropic and anisotropic analysis mode, with additional information of fibre diameter, bulk density and porosity. “Ave.” and “Stdv.” stand for average and standard deviation, respectively.

Table S7. Thermal conductivity obtained in isotropic and anisotropic analysis mode, with additional information of fibre diameter, bulk density and porosity. "Ave." and "Stdv." stand for average and standard deviation, respectively.

Sample Code	Fibre Diameter	Bulk Density	Overall Porosity	Thermal Conductivity (κ_{xyz})		Thermal Conductivity (κ_z) (OP-direction)		Thermal Conductivity (κ_{xy}) (IP-direction)	
	Ave. (μm)	(g cm^{-3})	(%)	(W m^{-1} K^{-1})	Stdv. (W m^{-1} K^{-1})	(W m^{-1} K^{-1})	Stdv. (W m^{-1} K^{-1})	(W m^{-1} K^{-1})	Stdv. (W m^{-1} K^{-1})
**Rayon soft CFs**									
**SFG1aC**	10.7	0.073	95.7	0.065	0.001	0.034	0.000	0.107	0.004
**SFC1aSI**	9.59	0.081	95.2	0.064	0.001	0.046	0.000	0.084	0.0156
**SFC1aC**	12.02	0.098	94.8	0.071	0.008	0.072	0.013	0.058	0.009
**SFG1aSI**	9.7	0.085	94.8	0.071	0.002	0.042	0.006	0.152	0.003
**SFG1aBG**	12.57	0.092	94.6	0.079	0.005	0.041	0.009	0.143	0.016
**SFC1aBG**	11.98	0.106	93.9	0.077	0.000	0.085	0.011	0.060	0.005
**SFG1aSC**	10.3	0.108	92.7	0.126	0.002	0.043	0.002	0.292	0.003
**PAN (thick fibres) soft CFs**									
**SFG2bC**	19.71	0.110	93.8	0.172	0.015	0.026	0.001	0.806	0.018
**SFG2bBG**	17.98	0.122	93.3	0.190	0.003	0.037	0.007	1.023	0.129
**SFC2bBG**	18.53	0.120	93.2	0.201	0.005	0.031	0.001	1.181	0.038
**SFC2bC**	18.69	0.140	92.5	0.103	0.005	0.094	0.009	0.103	0.008
**PAN (thin fibres) soft CFs**									
**SFC2aBG**	10.56	0.084	95.3	0.126	0.001	0.127	0.000	0.121	0.001
**SFG2aC**	9.06	0.091	95.0	0.238	0.006	0.038	0.005	0.810	0.154
**SFC2aZF**	9.05	0.116	93.4	0.194	0.005	0.126	0.031	0.349	0.015
**Rayon rigid boards**									
**RBG1aSI**	10.01	0.160	89.6	0.234	0.008	0.110	0.002	0.436	0.011
**RBG1aC**	11.63	0.200	87.5	0.270	0.010	0.042	0.007	1.791	0.118
**Rigidized PAN (thick fibres) soft CFs**									
**rSFG2bBG**	16.96	0.180	89.3	0.324	0.041	0.050	0.014	1.473	0.293
**rSFC2bC**	19.59	0.230	88.1	0.176	0.019	0.142	0.016	0.130	0.032

An experimental method already reported elsewhere [6] has been adapted to perform a comprehensive experimental study of the effect of compression on the thermal conductivity of CFs. A controlled load along the z-axis (out-of-plane direction) of the samples was obtained using the Instron 5944 universal testing machine (INSTRON, 825 University Ave Norwood, MA, USA) described earlier (see Figure S14). The compressive load was increased before each thermal conductivity measurement, and after each change in thickness, the sample was left for at least 30 minutes to stabilise before the thermal conductivity was measured again. This measurement was performed with the Hot Disk TPS 2500 (Hot Disk AB, Sven Hultins gata 9A 412 88 Göteborg, Sweden) device as described in Section 4.2, under the same fixed conditions of temperature (20 °C) and relative humidity (50%). The Hot Disk® sensor was installed on an height-adjustable sample holder (see Figure S14), which allowed it to be precisely positioned. Finally, the unit was placed behind a transparent cover that protected the sample being measured from draughts (see Figure S14). Each result is the average of at least three measurements. The results of the experimental measurements are presented in Table S8.

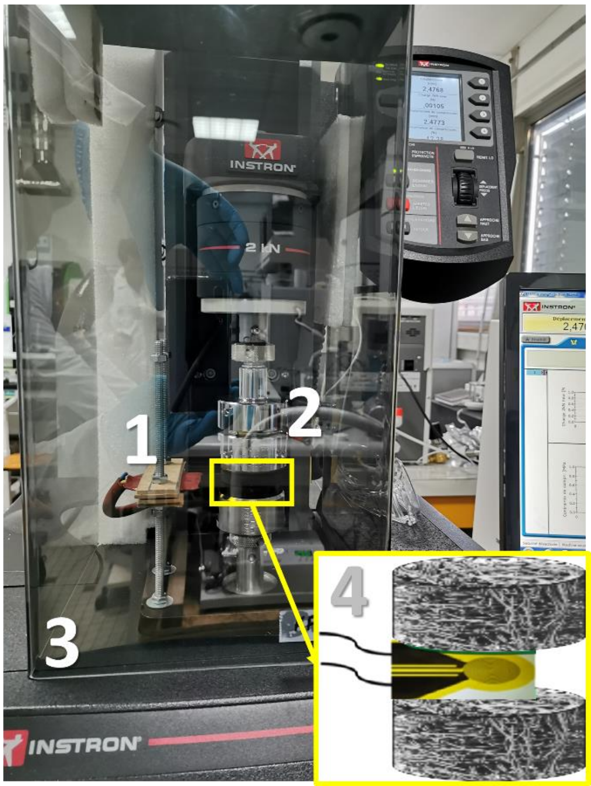

Figure S14. Experimental testing setup: 1—Hot Disk® sensor (connected to the Hot Disk® TPS 2500 device) installed between the upper and lower samples and fixed by a support made of insulating material and adjustable in height; 2—Compression platens of the Instron device; 3—Draught-free enclosure; 4—Schematic presentation of the CF sample set for testing.

Table S8. Thermal conductivity of Rayon-derived softs CFs subjected to compression, obtained in isotropic and anisotropic mode, with additional information on modified bulk density and porosity of the sample.

Compression Force		Bulk Density	Overall Porosity	Thermal conductivity (κ_{xyz})		Thermal conductivity (κ_{z}) (OP-direction)		Thermal conductivity (κ_{xy}) (IP-direction)	
(N)	Stdv. (N)	(g cm^{-3})	(%)	(W m^{-1} K^{-1})	Stdv. (W m^{-1} K^{-1})	(W m^{-1} K^{-1})	Stdv. (W m^{-1} K^{-1})	(W m^{-1} K^{-1})	Stdv. (W m^{-1} K^{-1})
**SFC1aSI**									
0.440	0.085	0.081	95.200	0.062	0.000	0.048	0.001	0.080	0.001
1.683	0.003	0.141	91.159	0.058	0.001	0.049	0.004	0.070	0.002
4.348	0.028	0.213	86.686	0.058	0.003	0.049	0.001	0.068	0.006
7.658	0.035	0.277	82.670	0.063	0.001	0.055	0.000	0.072	0.001
**SFG1aSI**									
0.503	0.057	0.085	94.800	0.057	0,002	0.027	0.005	0.121	0.002
1.249	0.200	0.156	90.583	0.063	0.001	0.028	0.001	0.144	0.007
2.012	0.156	0.226	86.360	0.067	0.001	0.029	0.005	0.159	0.010
4.867	0.188	0.252	84.777	0.071	0.001	0.032	0.000	0.157	0.001
**SFC1aBG**									
0.455	0.010	0.106	93.900	0.070	0.000	0.068	0.001	0.071	0.002
1.560	0.005	0.178	89.880	0.076	0.000	0.095	0.000	0.061	0.000
1.700	0.006	0.257	85.360	0.078	0.000	0.112	0.000	0.054	0.003
7.040	0.011	0.320	81.790	0.078	0.005	0.127	0.000	0.048	0.001
**SFG1aBG**									
0.414	0.006	0.092	94.600	0.085	0.001	0.052	0.001	0.139	0.001
1.913	0.010	0.171	90.522	0.089	0.000	0.059	0.000	0.133	0.001
4.316	0.005	0.251	86.044	0.092	0.000	0.067	0.000	0.126	0.000
11.617	0.005	0.323	82.060	0.094	0.001	0.070	0.000	0.126	0.003

Table S8 (continued)

Compression force		Bulk density	Overall porosity	Thermal conductivity (κ_{xyz})		Thermal conductivity (κ_z) (OP-direction)		Thermal conductivity (κ_{xy}) (IP-direction)	
(N)	Stdv. (N)	(g cm^{-3})	(%)	(W m^{-1} K^{-1})	Stdv. (W m^{-1} K^{-1})	(W m^{-1} K^{-1})	Stdv. (W m^{-1} K^{-1})	(W m^{-1} K^{-1})	Stdv. (W m^{-1} K^{-1})
				**SFC1aC**					
0.500	0.015	0.098	94.900	0.064	0.002	0.061	0.001	0.067	0.002
2.500	0.042	0.171	90.887	0.066	0.000	0.068	0.000	0.065	0.000
6.700	0.049	0.256	86.400	0.064	0.001	0.068	0.005	0.061	0.003
11.226	0.000	0.331	82.390	0.065	0.002	0.078	0.004	0.054	0.002
				**SFG1aC**					
0.450	0.002	0.073	95.700	0.051	0.000	0.026	0.000	0.099	0.001
0.593	0.004	0.110	94.200	0.053	0.001	0.030	0.001	0.096	0.002
0.685	0.001	0.129	93.200	0.054	0.000	0.032	0.000	0.093	0.001
1.053	0.042	0.158	91.675	0.055	0.000	0.034	0.000	0.089	0.001

References

1. Rocha, R.P.A.; Cruz, M.E. Calculation of the Permeability and Apparent Permeability of Three-Dimensional Porous Media. *Transp. Porous Media* **2010**, *83*, 349–373, doi:10.1007/s11242-009-9445-7.
2. Nouri, N.; Panerai, F.; Tagavi, K.A.; Mansour, N.N.; Martin, A. Evaluation of the Anisotropic Radiative Conductivity of a Low-Density Carbon Fiber Material from Realistic Microscale Imaging. *Int. J. Heat Mass Transf.* **2016**, *95*, 535–539, doi:10.1016/j.ijheatmasstransfer.2015.12.004.
3. Karakashov, B.; Toutain, J.; Achchaq, F.; Legros, P.; Fierro, V.; Celzard, A. Permeability of Fibrous Carbon Materials. *J. Mater. Sci.* **2019**, *54*, 13537–13556, doi:10.1007/s10853-019-03854-5.
4. Soltani, P.; Johari, M.S.; Zarrebini, M. Tomography-Based Determination of Transverse Permeability in Fibrous Porous Media. *J. Ind. Text.* **2015**, *44*, 738–756, doi:10.1177/1528083713512357.
5. Danes, F.; Bardon, J.-P. Conductivité Thermique Des Feutres de Carbone, Isolants à Forte Anisotropie: Modèle de Conduction Par La Phase Solide. *Rev. Générale Therm.* **1997**, *36*, 302–311, doi:10.1016/S0035-3159(97)80690-3.
6. Piégay, C.; Glé, P.; Gourdon, E.; Gourlay, E. A Cylindrical Self-Consistent Modelling of Vegetal Wools Thermal Conductivity. *Constr. Build. Mater.* **2020**, *232*, 117123, doi:10.1016/j.conbuildmat.2019.117123.
